# Early Boost of Linguistic Skills? Individualized Non-Invasive Brain Stimulation in Early Postacute Aphasia

**DOI:** 10.3390/brainsci14080789

**Published:** 2024-08-03

**Authors:** Ilona Rubi-Fessen, Kathrin Gerbershagen, Prisca Stenneken, Klaus Willmes

**Affiliations:** 1 Neurological Rehabilitation Hospital, RehaNova Köln, 51109 Cologne, Germany; gerbershagenk@rehanova.de; 2 Department of Rehabilitation and Special Education, Faculty of Human Sciences, University of Cologne, 50931 Cologne, Germany; prisca.stenneken@uni-koeln.de; 3 Department of Neurology, Medical Faculty, RWTH Aachen University, 52074 Aachen, Germany; willmes@gmx.de

**Keywords:** subacute aphasia, non-invasive brain stimulation (NIBS), transcranial direct current stimulation (tDCS), aphasia therapy, spontaneous recovery, language, Bayes factor

## Abstract

Non-invasive brain stimulation, such as transcranial direct current stimulation (tDCS), has been shown to increase the outcome of speech and language therapy (SLT) in chronic aphasia. Only a few studies have investigated the effect of add-on tDCS on SLT in the early stage of aphasia; this may be due to methodological reasons, in particular the influence of spontaneous remission and the difficulty of establishing stimulation protocols in clinical routines. Thirty-seven participants with subacute aphasia (PwA) after stroke (23 men, 14 women; mean age 62 ± 12 years; mean duration 49 ± 28 days) were included in two consecutive periods of treatment lasting two weeks each. During the first period (P1) the participants received 10 sessions of SLT, during the second period (P2) the aphasia therapy was supplemented by anodal left hemispheric 2 mA tDCS over the left hemisphere. Severity-specific language tests (Aachen Aphasia Test (AAT), *n* = 27 and Bielefeld Aphasia Screening-Reha (BIAS-R), *n* = 10) were administered before P1, between P1 and P2, and after P2. Where information was available, the results were corrected for spontaneous remission (AAT sample), and the therapy outcomes of P1 and P2 were compared. Participants’ overall language abilities improved significantly during P1 and P2. However, improvement—as measured by the AAT profile level or the BIAS-R mean percentage value—during P2 (with tDCS) was significantly higher than during P1 (*p* < 0.001; AAT sample and *p* = 0.005; BIAS-R sample). Thus, tDCS protocols can be implemented in early aphasia rehabilitation. Despite the limitations of the research design, which are also discussed from an implementation science perspective, this is preliminary evidence that an individually tailored anodal tDCS can have a significant add-on effect on the outcome of behavioral aphasia therapy in subacute aphasia.

## 1. Introduction

Aphasia as a neuropsychological language disorder is one of the most devastating consequences of stroke and can affect up to 40% of people after stroke [1,2,3]. Aphasia leads to reduced quality of life and social participation [4,5] and increased depression rates and mortality [1,6,7]. Aphasia usually persists chronically, and recovery is often prolonged and incomplete. Speech and language therapy (SLT) is the gold standard for supporting and enhancing spontaneous recovery in the early stages of aphasia. For chronic aphasia there is sound evidence that SLT can improve rehabilitation [8,9]. Although the acute and subacute stages of aphasia are a critical time for brain recovery [10], evidence for the effectiveness of speech therapy in the early stage is weaker and less convincing [11,12,13]. A promising approach to improving the efficacy of SLT might be methods of non-invasive brain stimulation (NIBS), such as (repetitive) transcranial magnetic stimulation (rTMS) and transcranial direct current stimulation (tDCS) as an adjuvant in behavioral aphasia therapy. There is converging evidence that NIBS can induce or enhance neuroplastic changes and support functional reorganization in aphasia [14,15,16], and be applied in combination with SLT to modulate the cortical excitability of different brain regions during language recovery after stroke. Both techniques can increase or inhibit cortical excitability depending on the particular stimulation paradigm. rTMS is a focal method with high spatial resolution that uses rapidly fluctuating magnetic pulses to induce or inhibit synaptic transmission and induce neuronal firing in a target region of the brain, but it requires extensive equipment and high-resolution imaging. In contrast, tDCS is an easy-to-apply, safe, and low-side-effect electrical stimulation of the brain, which modulates cortical excitability by applying weak electrical currents (typically 1–2 mA) to the scalp through a pair of surface electrodes (typically 5 × 7 cm^2^). It is assumed that the currents modulate neuronal resting membrane potentials and alter the likelihood of neuronal firing [17]. Depending on the placement of the anode or cathode electrode, tDCS can be applied in an excitatory or inhibitory mode [18,19].

There is emerging evidence for the effectiveness of anodal tDCS over the left inferior frontal gyrus on naming in chronic aphasia [14,16,20]. Small studies also report a positive effect of anodal tDCS over the motor cortex on naming and functional communication in contrast to sham stimulation in chronic aphasia [21,22]. The reactivation or recruitment of lesioned and perilesional left hemisphere regions involved in language-related tasks using anodal tDCS seems to be the most promising way for optimized recovery [18,23,24,25,26]. However, depending on, for example, lesion size, other stimulation areas are also conceivable, e.g., right-hemispheric areas [27].

Although neuroplasticity is greatest early after stroke and tDCS should be most effective in the subacute period, only a few studies have applied tDCS to persons in the early stage of aphasia, and the results concerning the additive effect of tDCS in this phase of aphasia are ambiguous. Spielmann et al. [28] found no effect of add-on anodal 1 mA stimulation for 20 min over the left inferior frontal gyrus (*n* = 26) in 10 sessions of a word-finding treatment compared to sham stimulation (*n* = 32) in persons with subacute aphasia. Stockbridge et al. [29] determined the stimulation site after observing activation in a naming task in fMRI or identified undamaged left hemisphere cortical areas using structural imaging. They detected an effect on recovery in discourse but not picture naming in a sample of 51 PwA. You and colleagues [30] compared the effect of SLT in combination with anodal tDCS over the left superior temporal gyrus (*n* = 7), cathodal tDCS over the right superior temporal gyrus (*n* = 7), and sham stimulation (*n* = 7) and observed significant progress in auditory speech comprehension after cathodal stimulation relative to the other groups.

Despite these inconsistent findings, the early phase after a stroke appears to be the crucial period for enhancing the beneficial neuroplasticity taking place in the damaged area and decreasing the maladaptive changes in compensatory areas by non-invasive brain stimulation [31]. Therefore, our aim was to deliver guideline-based and evidence-based treatment to achieve the highest level of linguistic performance. Although the primary goal of implementation science is the successful implementation of new evidence-based therapy methods and procedures rather than the actual outcome of the therapy method itself [32,33,34], it makes sense to integrate an implementation science perspective, particularly in communication sciences and disorders [35]. When planning our study for the early subacute phase, we were primarily faced with methodological challenges [31]. Reliable evidence of an additive benefit of tDCS in acute and subacute aphasia may be hampered by the difficulty of controlling for the influence of spontaneous remission, as the effects of therapy cannot be separated from the effects of spontaneous recovery. Also, to our knowledge, only two language tests for acute and subacute aphasia in German either have different/adapted norms for increasing days poststroke in acute aphasia [36] or allow a correction of the results for spontaneous recovery [37,38].

In addition, from the patients’ perspective, a standardized and uniform approach with respect to stimulation paradigms and behavioral aphasia therapy may not sufficiently consider the individual requirements and goals of patients in either the (early) subacute or chronic phases of aphasia [25,39,40].

Our aim, therefore, was to develop a methodologically controlled procedure based on the existing evidence that nevertheless leaves room for an individual decision about the choice of stimulation site/paradigms and therapeutic approach and that can also be integrated into the everyday clinical routine of a rehabilitation clinic. We intended to include patients with different types and severity of aphasia preferably within the (early) subacute phase of aphasia and to offer them targeted speech–language therapy and tDCS adapted to their individual symptomatology and needs. In the present study, we applied this protocol to a sample of PwA to evaluate the feasibility and effect of an adjuvant, individualized tDCS on the outcome of SLT. Our hypothesis was that two weeks of individualized stimulation over preserved tissue—thereby engaging the entire left hemispheric language network—combined with multimodal language therapy would lead to an increase in linguistic performance in PwA beyond what can be accomplished by language therapy alone.

## 2. Materials and Methods

### 2.1. Study Design

Participants underwent two consecutive two-week therapy treatment periods (P1 and P2) with 10 sessions SLT of 45 min each. SLT was combined with online anodal tDCS (20 min, 2 mA) over the left hemisphere (left IFG, left M1, or left FPC) during P2. Severity-specific linguistic assessments were administered just before P1, between P1 and P2, and immediately after P2. We compared the change in language performance between P1 and P2. P1 without tDCS served as the control condition.

### 2.2. Participants

The current study recruited participants from the neurorehabilitation clinic RehaNova Köln gGmbH between May 2019 and January 2024. Patients were suggested for screening by the treating speech–language pathologist (SLP) or physician. Inclusion criteria were unilateral left middle cerebral artery stroke, subacute aphasia as defined by Bernhardt et al. [41] (from day 7 to month 6, which can be subdivided into an early subacute phase (day 7 to end of month 3) and a late subacute phase (month 4 to end of month 6)), right-handedness, as measured by the Laterality Questionnaire by Salmaso and Longoni [42], testability, ability to follow simple verbal commands, and an attention span of about 60–90 min. Exclusion criteria were prior cerebrovascular stroke with language impairment, neurodegenerative or psychiatric disease, EEG with epileptiform activities or seizure patterns, and auditory or visual deficits that might impair assessment.

In total, 44 participants were screened for inclusion and exclusion criteria (see flow diagram in Appendix E). Two PwA declined to participate and two PwA had to be excluded because of severe limb apraxia that prevented them from following commands such as pointing at pictures. Forty participants were eligible for inclusion in the study, but three PwA were discharged from hospital shortly after language assessment 1. A total of 37 participants (23 men, 14 women; mean age 62 ± 12 years; mean duration 49 ± 28 days) completed the study protocol.

The PwA or their caregivers gave their written informed consent prior to participating in the study. This was performed in accordance with the ethical standards of the 2008 Declaration of Helsinki and with the approval of the local institutional review board of RehaNova (April, 2019). Table 1 provides an overview of the demographic and clinical parameters of these patients.

### 2.3. Clinical Assessment

#### 2.3.1. Language Assessment

Depending on participants’ severity of aphasia and verbal-expressive abilities, they were assessed with one of two standardized German language tests, with subtests measuring similar expressive and receptive language functions.

(1) Participants with the ability to communicate verbally, at least at a basic level (*n* = 27), were assessed with the Aachen Aphasia Test (AAT) (for a summary presentation of test properties in English, [37]). The AAT includes an evaluation of spontaneous language production on 6-point rating scales and 5 subtests: Token Test (TT), repetition (REP), written language (WRIT) (reading aloud, composing words and sentences from anagrams, writing to dictation), and naming (NAM), as well as auditory and written comprehension (COMP). The AAT allows for a probabilistic decision about the presence of aphasia (yes/no) and a probabilistic assignment to one aphasic type/syndrome, such as global aphasia, Wernicke’s aphasia, Broca’s aphasia, and anomic aphasia. The pattern of severity of impairments is provided in a subtest performance profile with subtest raw scores transformed into T-norm standardized scores for intersubtest comparisons of severity among language functions (T-scores based on a normative sample of 376 PwA). An overall estimate of severity of aphasia is given by the reliability-weighted average T-score across all AAT subtests (the so-called “profile level”). Syndrome-specific estimates for the expected spontaneous recovery from AAT assessments at 1, 4, and 7 months postonset in a historical control study by Willmes and Poeck [43] (see also Luzzatti et al. [44], Supplementary Materials) are available. Based on these data, correction factors were computed for the expected non-linear recovery across each of the two treatment periods P1 and P2 for the T-score profile level and the individual subtest raw scores (see Appendix C for details). Psychometric single-case analysis procedures (see Willmes [45]) were used to test for significant improvement in profile level and individual subtest performances for each individual PwA. A correction for expected spontaneous recovery in profile level and subtest performance can also be included (see Luzzatti et al. [44] for an example). At the group level, observed differences in performance across a therapy period were tested against zero as well as against these correction factors (listed in Appendix A Table A1) to be able to ascertain “pure” treatment effects.
(2) Participants with severe aphasia or without deliberate verbal-expressive communication skills (for example, due to severe apraxia of speech) were assessed with the Bielefeld Aphasia Screening Rehabilitation (BIAS-R) [46]. The BIAS-R is a linguistic assessment of aphasic impairments for persons with subacute aphasia, but unlike the AAT, it allows cues to provoke and support verbal responses in various subtests. The BIAS-R includes an optional analysis of spontaneous speech and eight subtests: auditory comprehension (ASV), automatic language use (AUT), naming (ELIZ), verbal fluency (WFL), repetition (REP), reading comprehension (LSV), reading aloud (LLES), and writing of words (WRIT). The overall severity of aphasia is determined by the average of the individual subtest percentage-of-maximum-value attained score. This average “raw” score can be transformed into a percentile rank or T-score based on a normative sample of 104 PwA. The test’s authors also provide critical differences for each subtest and the mean percentage value to identify (treatment-related) changes in performance that are probably beyond measurement error for the individual PwA.

Primary outcome measures, then, were the profile level (AAT) and the mean percentage value T-score (BIAS-R). Secondary outcome measures were all subtest raw scores and the spontaneous speech ratings of the AAT and the subtest percentages of the BIAS-R. The language tests were administered by one examiner (IRF). Because blinding was not possible due to established routines in the clinic, all verbal responses were digitally recorded and transcribed and analyzed by an experienced and trained research assistant who was blinded to treatment condition.

#### 2.3.2. Overall Disability and Side Effects

The degree of overall disability was determined by the Early Rehabilitation Barthel Index (ERBI) [47] and the Functional Independence Measure (FIM) [48], which is an internationally approved proxy-rating scale. In addition, side effects experienced after each stimulation were recorded on a visual analog scale by the PwA.

The ERBI rating was carried out by the ward physician, and the FIM rating was performed by clinical employees (nursing staff) who were not involved in the study.

### 2.4. tDCS Treatment

Transcranial direct current stimulation (tDCS) was applied using a battery-driven stimulator (DC-Stimulator mobile, NeuroConn GmbH, Ilmenau, Germany) with a pair of surface-soaked sponge electrodes (5 × 7 cm^2^ anode and 7 × 10 cm^2^ cathode). A constant current of 2 mA was applied at the beginning of the behavioral therapy for 20 min according to current safety recommendations [49].

Based on the CT/MRI diagnosis and the concordant judgment of two investigators (KG and IRF), three different electrode stimulation positions/montages were used: (1) If (re)activatable perilesional tissue in the left inferior frontal gyrus (left IFG) was preserved, anodal stimulation was applied over this area (Broca’s area, left IFG), corresponding to F5 of the international 10–20 EEG system; (2) in the case of extended lesions in the left IFG, which in all cases (*n* = 3) were associated with an extensive lesion of the superior temporal gyrus (STG), the anode was placed over the left primary motor cortex (M1), corresponding to C3 of the international 10–20 EEG system, to address the language network via its functional connections with the primary motor system [50,51,52]; (3) for three participants who had extensive lesions in the left IFG and left STG and had undergone cranioplasty after decompressive craniectomy, the anode was placed over the left frontopolar prefrontal cortex (FPC), corresponding to FP1 of the international 10–20 EEG system, to avoid stimulation over the scar and prevent the deviation of the current underneath the scar that was above the areas of the left IFG and the left STG [53]. In all conditions, the reference electrode was placed contralaterally over the right supraorbital region with a minimum distance of 10 cm from the anode. The larger size of the reference electrode was chosen to avoid bilateral bipolar stimulation by reducing the current flow density underneath the cathode [54].

After each stimulation, participants were asked to rate side effects (pain/discomfort) on a visual analog scale from 0 (no discomfort/pain) to 10 (high discomfort/pain). The SLPs recorded other side effects such as reddening of the skin.

The first SLT session with tDCS was carried out jointly by the examiner and the treating SLP to observe any side effects and give the PwA a feeling of security. The subsequent therapies were carried out by the treating SLP. All SLPs in the clinic were familiarized with the handling of tDCS through intensive personal training and an instruction manual.

### 2.5. Behavioral Therapy

Participants received individualized speech therapy tailored to their needs by experienced SLPs. Linguistically orientated multimodal therapy was applied, as this approach addresses specific linguistic functions that stimulate functional neural networks [55,56]. The specific content of the behavioral therapy was determined after language assessment 1 between the examiner and the treating SLP, considering the needs of the participants. Due to the early stage of aphasia and the possible fluctuation in clinical manifestations, short-term goals and medium-term communicative goals were primarily formulated for the duration of the inpatient stay. Whenever possible, these were developed together following the principle of collaborative goalsetting with the participants [57,58]. If this was not possible, information from relatives or caregivers was accessed, e.g., certain preferences, wishes, and topics (such as preferred food or drink, family and social environment, leisure activities, occupation). In addition to the needs of the patients in all linguistic modalities, the specific choice of items was based on linguistic criteria such as frequency (high- or low-frequency items), syntactic complexity (complex syntax or reduced syntactic structures [59]), and phonological structure for expressive tasks and/or in the presence of apraxia of speech. For the severely affected participants, the therapy was based on everyday language comprehension and verbal-expressive recall of high-frequency and familiar words and phrases. For the less severely affected PwA, the focus was on deliberate verbal and written word and sentence retrieval as well as advanced auditory and visual language comprehension. To guarantee comparability, for each participant the methodology and focus of the behavioral therapy were maintained across P1 and P2 and only adapted to the severity of the language impairment. The content of each SLT session was documented by the treating SLP.

### 2.6. Statistical Analyses

All descriptive and inferential statistical analyses were carried out using JASP software (version 0.18.3), including the preparation of graphical material [60]. For the AAT sample, initially repeated-measures ANOVAs with the factor “time” (3 levels) were carried out for the primary outcome measure T-score profile level as well as the average spontaneous speech rating and all subtest raw scores as quantitative secondary measures. Analogously for the BIAS-R sample, the same type of analysis was undertaken for the average percentage value T-norm score as the primary outcome measure and all subtest percentage values as quantitative secondary measures. Post hoc comparisons were performed with the Holm correction for multiple testing.

More directly related to the research hypotheses, one-tailed *t*-tests were applied to the differences in performance for therapy period 1 (P1) and therapy period 2 (P2) separately (and combined), as well as the difference of differences between periods 1 and 2. This latter analysis constitutes a direct test of the hypothesized superiority of the combined SLT and tDCS treatment. In addition to the test itself, effect sizes were determined using Cohen’s d, including the one-sided 95% confidence interval (95%-CI) for the parameter. The frequentist statistical analyses were augmented by computing the Bayes factor (BF_+0_ or BF_0+_ employing the default Cauchy prior with = 0.7) providing a degree of evidence for the respective alternative or null hypothesis. The latter is particularly helpful when trying to provide evidence for the actual truth of the null hypothesis, expressing the absence of a treatment effect. The degree of evidence is classified (in accordance with the criteria implemented in JASP) as either anecdotal (1 ≤ BF < 3), moderate (3 ≤ BF < 10), strong (10 ≤ BF < 30), or very strong (BF ≥ 30). In the case of major violations of the normality assumption for the difference scores, in particular when there is one or a few outlying values, the Wilcoxon signed rank test was employed; for this test an effect size measure is available in JASP, but there is no BF analysis. Correlations were computed using Pearson product moment correlation coefficients or Spearman rank correlations.

Although the sample sizes are not large, a “sensitivity” analysis undertaken with the power analysis software G*Power 3.1 (version 3.1.9.2; Faul et al., 2009 [61]) revealed for the one-sided one-sample *t*-test improvement in performance over a treatment period, for which an effect size (Cohen’s d) of d = 0.65 (0.49) can be detected for α = 0.05, β = 0.95 (0.80). For the smaller BIAS-R group sample size of *n* = 10, the corresponding effect size values are d = 1.13 (0.85).

## 3. Results

All included participants completed the study protocol. Because the participants were assigned to separate groups based on the severity of the speech-related symptoms and were assessed with different aphasia tests, both groups (AAT sample, *n* = 27; BIAS-R sample, *n* = 10) were evaluated separately for their treatment outcomes (see Appendix D Table A4 and Table A5 for more detailed results). Prior to assessment, the two groups were analyzed for comparability in sociodemographic and basic neurological aspects (see Appendix D Table A3 for individual patients’ characteristics).

### 3.1. Group Comparability

Although there were more men than women in the AAT sample, the gender distribution did not differ significantly (χ^2^(1) = 0.59, *p* = 0.44, with continuity correction (cc)). Age (t(35) = 0.80, *p* = 0.43, Cohen’s d = 0.29 [−0.44–1.02], BF_01_ = 2.27) and years of education (t(35) = 0.44, *p* = 0.67, Cohen’s d = 0.37 [−0.57–0.89], BF_01_ = 2.68) were also comparable. The BIAS-R sample had a significantly longer duration (t(35) = 2.32, *p* = 0.027, d = 0.84 [0.08–1.59], BF_10_ = 2.45) and was in general more severely impaired, as indexed by the ERBI score (t(35) = 5.82, *p* < 0.001, d = 2.11 [1.22–2.97], BF_10_ = 8753) and the FIM score (t(35) = 2.90, *p* = 0.006, d = 1.05 [0.28–1.81], BF_10_ = 6.93). A total of 24/27 PwA in the AAT sample were therefore in the early subacute phase (day 7 to the end of month 3) according to the Bernhardt et al. [41] classification, in contrast to 6/10 PwA in the BIAS-R sample (see Appendix D Table A3 for details). 

For the BIAS-R sample, the overall gross aphasia severity grading was based on the BIAS-R average percentage value stanine (SN) score with the following levels: severe = SN 1–3, moderate = SN 4–5, mild = SN 6–7, and residual = SN 8–9. For the AAT sample, this overall gradation employed the AAT profile level SN grading with the same four levels. A chi-square contingency table test revealed that the overall aphasia severity was significantly stronger in the BIAS-R sample (χ^2^(2) = 8.33, *p* = 0.040, with cc). As regards fluency of speech output, the BIAS-R sample contained almost exclusively PwA with non-fluent output, while there were more PwA of the fluent type in the AAT sample (χ^2^(1) = 6.21, *p* = 0.013, with cc). Stimulation site for the AAT sample was almost exclusively over the IFG, whereas the sites were more varied for the BIAS-R sample (IFG, M1, FPG) (χ^2^(2) = 12.36, *p* = 0.002, with cc).

### 3.2. Primary Outcome Measure

**Figure 1:**
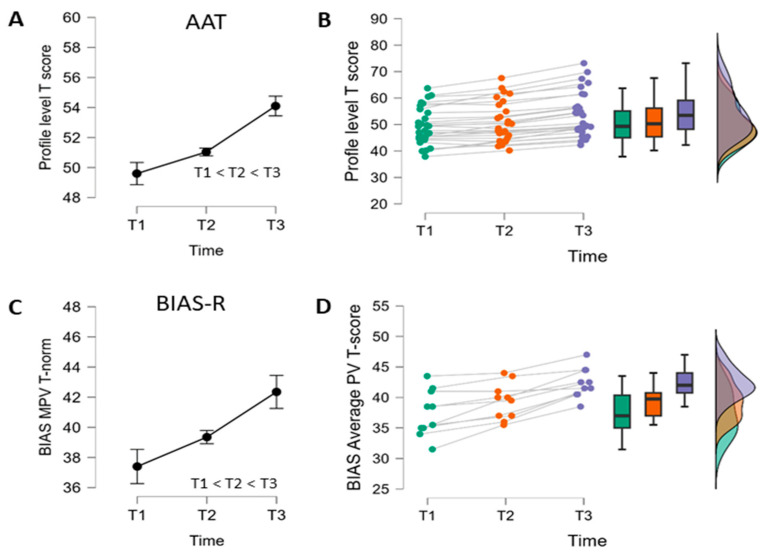
Global primary outcome measure analysis. (**A**,**C**): Repeated-measures ANOVA, cell means and within-subject 95%-CI (Morey, 2008) [63] as well as result of Holm-corrected comparison of assessments for AAT profile level or BIAS-R mean percentage correct T-score; (**B**,**D**): Raincloud plots of individual performance courses including boxplots and density estimates.

The three language assessments, taken at pretreatment (T1), post-SLT period 1 (T2), post-SLT + tDCS treatment period 2 (T3), for the two primary profile level outcome measures yielded a strong overall effect across time with significant improvement in performance for both therapy periods separately (see Table 2 and Figure 1). As the raincloud plots for the individual courses of performance across therapy show, the improvements in performance look quite homogeneous with only minor crossings of individual courses, if present at all. The statistical findings were qualitatively the same when discarding the one PwA with FPC as the stimulation site.

**Table 2:** Descriptive information (arithmetic mean (M), standard deviation (SD)) and inferential statistical analysis of performances across the therapy period; A: AAT profile level T-score, average spontaneous speech rating, and subtest raw scores; B: BIAS-R average percentage value T-score and subtest percentage values.

**A**	**AAT**	**T1**	**T2**	**T3**	**ANOVA Factor Time**			**Post Hoc** **Compar. ^§^**
	**(*n* = 27)**	**M (SD)**	**M (SD)**	**M (SD)**	**F(2,52) ^#^**	***p***	**η^2^_p_**	
	Profile level (T-norm)	49.60 (7.02)	51.04 (7.50)	54.10 (8.50)	63.97	<0.001	0.71	T1 < T2 < T3
Spont.	Ave. spont. speech rating	2.83 (1.91)	2.95 (0.82)	3.27 (0.82)	14.08	<0.001	0.35	(T1,T2) < T3
TT	Token Test (errors)	27.78 (16.10)	24.04 (16.24)	19.00 (15.67)	25.37	<0.001	0.49	T1 < T2 < T3
REP	Repetition	102.70 (37.80)	107.82 (33.68)	117.82 (28.77)	26.98	<0.001	0.51	T1 < T2 < T3
WRIT	Written language	39.44 (26.68)	44.34 (26.76)	50.56 (26.85)	33.64	<0.001	0.56	T1 < T2 < T3
NAM	Naming	51.74 (35.04)	56.70 (36.71)	67.15 (35.39)	25.41	<0.001	0.49	T1 < T2 < T3
COMP	Comprehension	77.33 (18.30)	79.59 (19.66)	89.11 (17.44)	26.64	<0.001	0.51	(T1,T2) < T3
**B**	**BIAS-R**	**T1**	**T2**	**T3**	**ANOVA Factor Time**			**Post Hoc** **Compar. ^§^**
	**(*n* = 10)**	**M (SD)**	**M (SD)**	**M (SD)**	**F(2,18) ^#^**	***p***	**η^2^_p_**	
	Profile level (T-norm)	37.40 (3.81)	39.35 (2.97)	42.35 (2.45)	35.55	<0.001	0.80	T1 < T2 < T3
ASV	Auditory comprehension	63.91 (12.57)	69.33 (11.26)	75.95 (9.35)	8.64	0.002	0.49	(T1,T2) < (T2,T3)
AUT	Automated sequences	42.98 (15.98)	52.22 (18.39)	66.90 (17.86)	29.02	<0.001	0.76	T1 < T2 < T3
ELIZ	Confrontation naming	40.80 (6.94)	41.37 (7.11)	48.31 (6.31)	3.85	0.041	0.30	(T1,T2,T3)
NACH (*n* = 9)	Repetition	28.88 (25.39)	36.30 (26.12)	61.09 (23.57)	13.92	<0.001	0.64	(T1,T2) < T3
LSV	Reading comprehension	58.49 (18.94)	63.79 (20.00)	69.54 (19.59)	17.24	<0.001	0.66	T1 < T2 < T3
LLES	Reading aloud	24.97 (26.14)	33.28 (24.01)	41.32 (27.51)	6.09	0.010	0.40	(T1,T2) < (T2,T3)
SCHR	Written language	8.33 (12.35)	16.60 (19.80)	27.73 (22.83)	6.61	0.007	0.42	(T1,T2) < (T2,T3)

^#^ Adjustment of df in case of lacking compound symmetry according to Geisser and Greenhouse. ^§^ Pairwise comparisons and adjustment of *p*-values for the sequentially rejective multiple test procedure by Holm (1979) [62] for a family of three tests, overall type-I error level = 0.05; time points in brackets do not have significant differences in performance.

**Figure 2:**
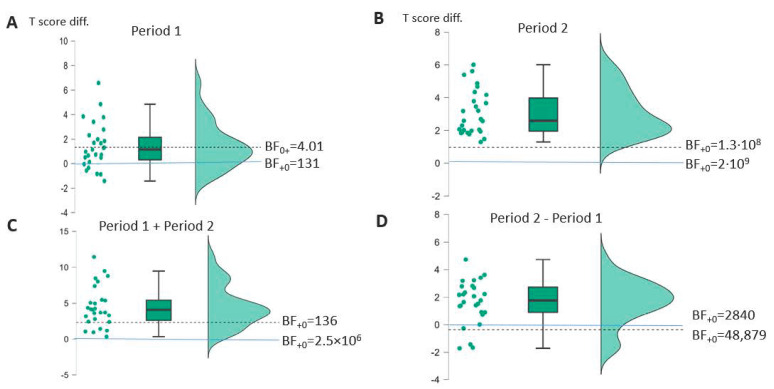
Raincloud plots of AAT profile level changes in T-scores (**A**): Across period 1, (**B**): Across period 2, (**C**): Across periods 1 + 2, (**D**): Across period 2 vs. period 1; Bayes factors for one-sided *t*-test against expected spontaneous recovery change (dashed line) and no (zero) change (solid line).

**Figure 3:**
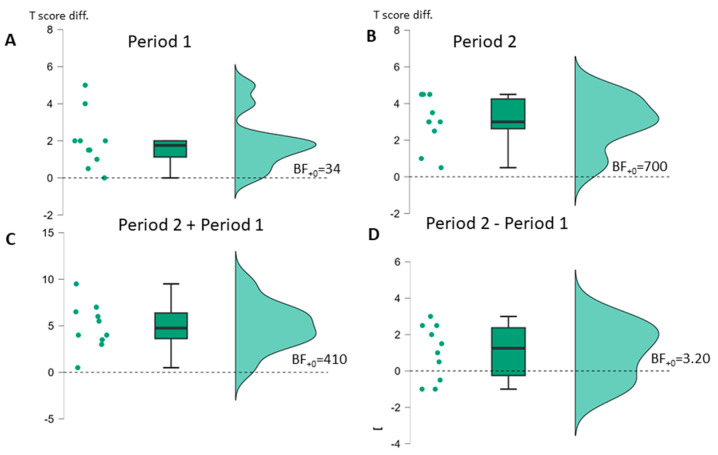
Raincloud plots of BIAS-R average percentage value T-score changes (**A**): Across period 1, (**B**): Across period 2, (**C**): Across periods 1 + 2, (**D**): Across period 2 vs. period 1; Bayes factor for one-sided *t*-test against no change (dashed line).

More informative for the research questions of this study are the analyses of the post-minus pretherapy period difference scores tested one-sided against zero (no effect of therapy) separately with a *t*-test for both periods and the direct comparison of the difference score for P2 minus the difference score for P1, tested one-sided against zero (no difference in effect of therapy for SLT + tDCS vs. SLT alone) with a *t*-test as well (see Table 3 and Figure 2 and Figure 3). For the AAT group, it was possible to test these differences more adequately not against zero but against the expected estimated difference attributable to spontaneous recovery, as computed from the historical spontaneous recovery data available for the AAT [43,44] (see Appendix C for details of the estimation procedure). Since spontaneous recovery data are not available for the BIAS-R, the information for tests against a difference of zero is provided for the AAT group (see Appendix A Table A2).

For the AAT profile level in therapy P1, there is no significant improvement beyond what can be expected from spontaneous recovery, with the Bayes factor indicating moderate evidence for the null hypothesis of no change beyond spontaneous recovery. When testing the change in AAT profile level against zero, however, there is a strong effect with strong evidence that the improvement is true (see Appendix A Table A2 first row).

The situation is different for P2 with added tDCS stimulation. The average difference score is significantly larger than would be expected according to spontaneous recovery, with a large Cohen’s d effect size estimate and very strong evidence that the alternative hypothesis of positive change is true (see Table 3A). Cohen’s d is well above the limit of 0.65 from the sensitivity analysis, even the lower bound of its confidence interval is larger than that value. As the raincloud plot in Figure 2B nicely reveals, all PwA in the AAT group had a positive change in performance beyond the expected spontaneous recovery value (dashed line). Moreover, the improvement after the combined therapy was significantly higher than after SLT alone (see Figure 2D and Table 3A), with only three patients revealing numerically higher changes in P1. Again, Cohen’s d is above the threshold of 0.65 from the sensitivity analysis. Furthermore, there was a large significant improvement in performance beyond expectation due to spontaneous recovery across the whole treatment phase of four weeks (see Figure 2C and Table 3A), the effect estimate again being beyond the threshold from the sensitivity analysis.

For the BIAS-R group, a similar pattern of effects was present (see Figure 3 and Table 3B) for all one-sided *t*-tests against the null hypothesis of no change. Here, also in P1—with no information regarding spontaneous recovery effects available—a moderate to strong improvement was found according to the 95%-CI for Cohen’s d, with the effect size estimate beyond the critical value of 1.13 for both periods individually and for the combined period. The differential superiority effect of the combined treatment was also present (numerically for 7 out of 10 PwA), but less strong than for the AAT group and providing just moderate evidence that the alternative hypothesis is true. The statistical results were very similar when introducing the grouping factor stimulation site, with no significant main effect of stimulation site nor its interaction with time (both F < 1).

When additionally testing all changes in performance against zero for the AAT group, all *t*-tests revealed strong effects and very strong evidence for the alternative hypothesis, as well as for superiority of the combined therapy, in particular (see also Table A2).

Another perspective on treatment success is looking for the number of “responders”, i.e., PwA whose individual difference score is above the critical difference for a reliable improvement in performance. For the AAT, this critical value (at a type-I error level of 10%) is 1.10 T-scores [37]. In the first therapy period, 13/27 = 48.15% of the PwA improved significantly; this proportion was 100% for the second period with the combined treatment. Additionally, 19/27 = 70.37% improved significantly in the second period compared to the first.

For a more adequate comparison with the AAT, the reported critical difference for the BIAS-R was computed for a type-I error level of 10% to a 9.2 change for the average percentage value. In the first therapy period, 1/10 of the PwA improved significantly; in the second period, 6/10. The PwA with significant improvement during P1 increased their gains by more than a third during P2. The exact one-sided binomial test revealed a significant increase (*p* = 0.03125) in the number of responders from period 1 to period 2 for the BIAS-R overall performance level.

### 3.3. Secondary Outcome Measures

All statistical analyses for the primary outcome measures were also carried out for the individual subtests from the AAT, including the average spontaneous speech rating core, and from the BIAS-R, only the word fluency subtest, for which the PwA showed consistent floor effects, was left out. As is usual in (more exploratory) secondary analyses, the type-I error level was kept at 0.05 for each subtest, but the respective results tables in Appendix B always report *p*-values such that the Bonferroni or Holm corrections could be made easily.

**Figure A1:**
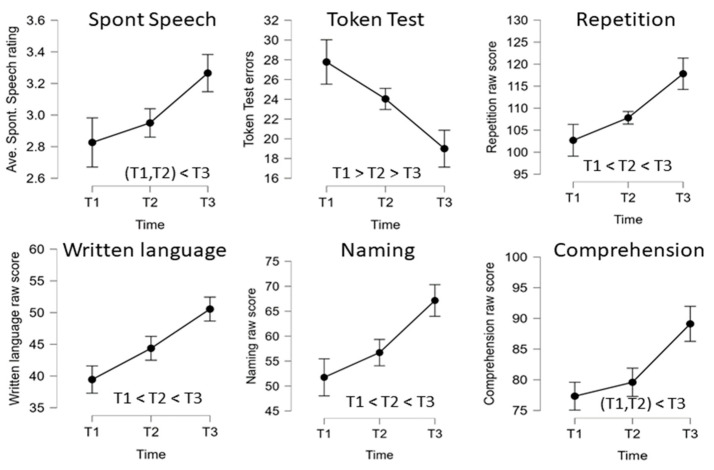
Repeated-measures ANOVAs for AAT average spontaneous speech rating and subtest raw scores: Cell means and within-subject 95%-CI as well as Holm-corrected comparison of assessments.

**Figure A2:**
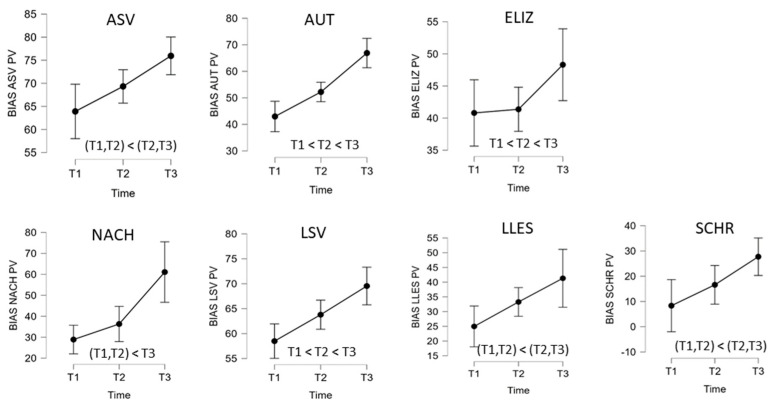
Repeated-measures ANOVAs for BIAS-R subtest percentage values: Cell means and within-subject 95%-CI as well as Holm-corrected comparison of assessments.

**Figure A3:**
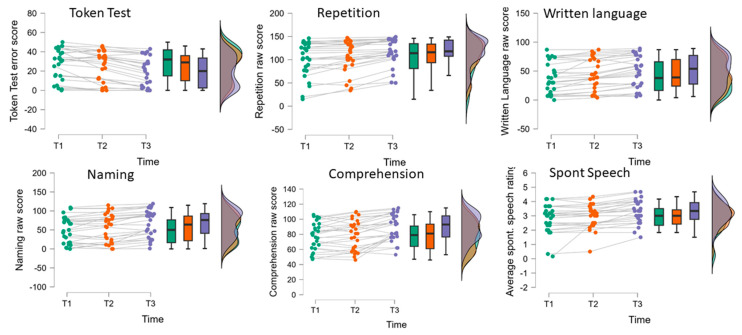
Raincloud plots of individual AAT average spontaneous speech rating and subtest raw score performances across the treatment period.

Again, there is the impression of a strong time effect in the repeated-measures ANOVAs for subtests across all three assessments, as visualized in Figure A1 for the AAT and Figure A2 for the BIAS-R. The prevailing pattern for the AAT is a significant increase in performance for each period except for the subtest comprehension and the average spontaneous speech rating, for which the increase in P1 was only numerical but not significant after being Holm-corrected for the pairwise comparisons of time points. Figure A3 shows the individual courses of AAT subtest performances across all three assessments. The overall impression is a regular, more or less steep increase with only a few crossings, sharp rises, or declines in one period (e.g., the latter two course types are most obvious for subtest comprehension).

**Figure A4:**
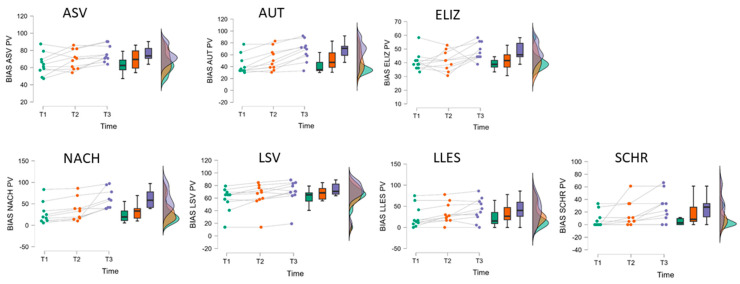
Raincloud plots of individual BIAS-R subtest percent correct value (PV) performances across the treatment period.

For the BIAS-R, with its small sample size, the prevailing patterns were either a significant increase for each therapy period (subtests AUT, ELIZ, LSV) or again an intermediary position at T2, which was, however, not significantly different from either performance at T1 or T3 (subtests ASV, LLES, SCHR). Figure A4 shows the individual courses of BIAS-R subtest performance across all three assessments. The overall impression is a regular, more or less steep increase with only a few crossings, sharp rises, or declines in one period (e.g., in particular for subtests ASV, ELIZ), and steeper increases in period 2 (e.g., for subtests NACH, LLES, SCHR).

When inspecting the ANOVA results in Table 2A, the overall effect size estimate (partial eta^2^) is large for all AAT subtests (Cohen, 1988 [64]; also see the discussion by Lakens [65] for the interpretation of ANOVA effect sizes), ranging from 0.35 for the average spontaneous speech rating up to 0.56 for subtest written language. Likewise, there are large effect size estimates for the BIAS-R, but the small sample size must be taken into consideration, since even for ELIZ the effect size estimate—although the *p*-value is just 0.041—is quite high. The strongest effects ranging from 0.64 to 0.76 were present for NACH, LSV, and AUT, in line with the results of the pairwise comparisons and the impression from Figure A2.

**Figure A5:**
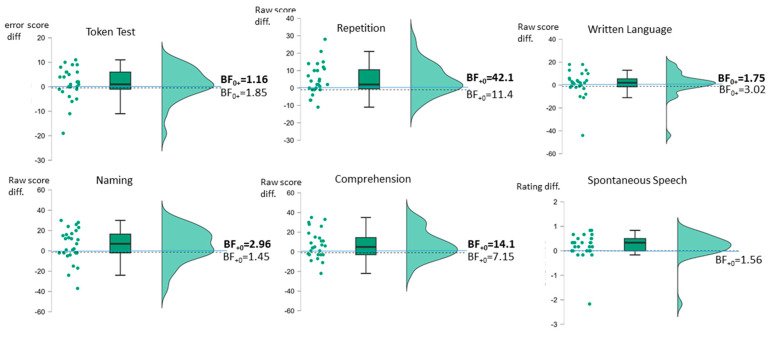
Raincloud plots of AAT individual subtest differential changes for period 2 -period 1; Bayes factors (bold font) for one-sided *t*-test against expected spontaneous recovery change (dashed line) and (regular font) against no (zero) change (solid line).

The more detailed analyses for changes in performance per therapy period (see Table 3A) for the AAT subtests consistently revealed that across all subtests there is no significant improvement beyond what might be expected due to spontaneous recovery, with anecdotal to moderate evidence for the null hypothesis of no improvement holding. The situation was very different for the second treatment period. There was significant improvement for all subtests (all *p* = 0.004 or less and are thus well below the Holm adjustment for a family of five tests) with moderate to mostly strong effect sizes and strong to very strong evidence for the alternative of improvement to hold. The analysis for differentially more improvement after the combined treatment yielded significant effects for the repetition, comprehension, and naming subtests (the latter only in the case of no Holm adjustment). The first two had medium effect sizes, providing strong evidence for a true superior increase in performance after the combined treatment. The raincloud plots in Figure A5 illustrate the varying degrees of differential improvement. For the whole therapy period of four weeks there was significant improvement (also after Holm adjustment) with medium to strong effect sizes and strong to very strong evidence for true improvement.

For the AAT, average spontaneous speech rating improvement (tested against zero) was only significant for period 2 and the whole therapy phase of four weeks (see Appendix A Table A2), both with a strong effect and strong evidence that the improvement is a true effect.

**Figure A6:**
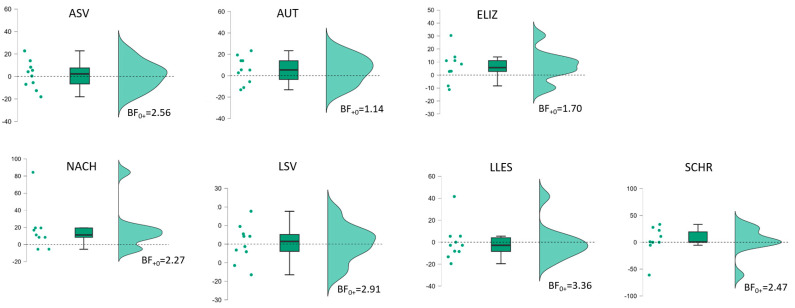
Comparison of period 2 vs. 1 for BIAS-R subtest percentage values: Bayes factor for one-sided *t*-test against zero.

A parallel, more detailed analysis for changes in performance per therapy period was also carried out for the BIAS-R subtests (see Table 3B). Significant and large improvement effects were present for AUT, NACH, LSV, and LLES, with mostly strong evidence for the alternative hypothesis of true improvement. In the second period with add-on tDCS treatment, all subtests except for LLES revealed significant improvement, with moderate to strong effects size estimates and moderate to strong evidence of true improvement. Somewhat differently from the AAT group, there was no compelling superiority of the combined treatment over the SLT in the first period when adjusting for multiple testing. Only the repetition subtest NACH had a *p*-value of less than 0.05 individually. This lack of an effect can also be seen from the raincloud plots in Figure A6. For the whole four-week therapy period, we found significant improvement even after using the Holm adjustment for all subtests except for LLES and ELIZ, with strong effect sizes and strong to very strong evidence for true improvement. For LLES the evidence was only moderate and for ELIZ anecdotal.

The level of discomfort/pain caused by the stimulation across all sessions was rated as mild on a scale of 0–10. Whole sample (*n* = 37) mean = 1.67 (SD 1.886); IFG (*n* = 31) mean = 1.74 (SD 1.991); M1 (*n* = 3) mean = 0.07 (SD 0.115); FPC (*n* = 3) mean = 2.3 (SD 0.781) (see Appendix D Table A6).

**Figure A7:**
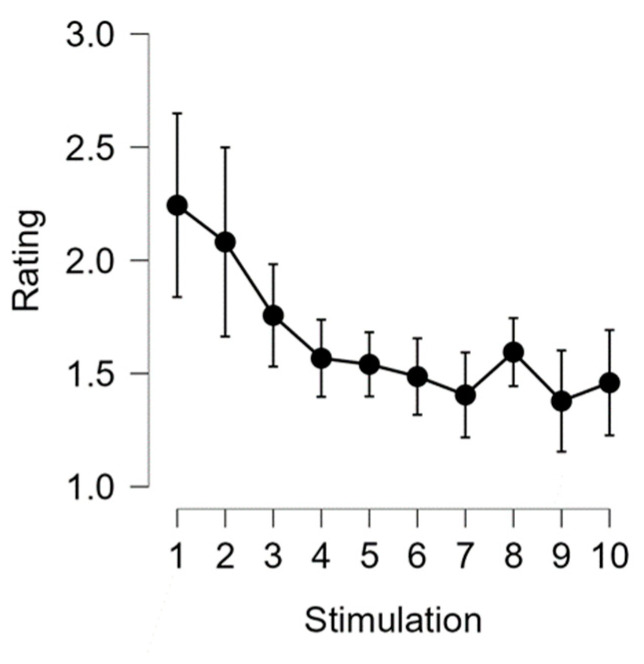
Course of side effect severity ratings for all PwA (*n* = 37).

Figure A7 shows the average course of side effect severity ratings for all PwA, which decreased over the series of 10 stimulation occasions. The initial level of side effect severity ratings was already low and decreased continuously down to about 1.5. A repeated-measures ANOVA indicated a significant effect (F(9,324) = 5.57, *p* < 0.001) and subsequent polynomial trend analyses revealed significant linear and quadratic trend components (both *p* < 0.001).

### 3.4. Correlational Analyses

Initial performance on the AAT and the BIAS-R was not correlated significantly with age or duration postonset, nor with the ERBI or the FIM score.

**Figure 4:**
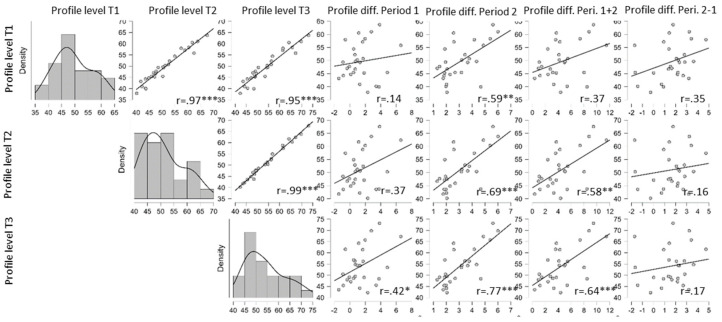
Correlations among level of and change in performance for the AAT profile level for the three assessments; * *p* < 0.05, ** *p* < 0.01, *** *p* < 0.001.

Figure 4 shows scatter plots and correlation coefficients among the AAT profile T-scores for the three individual assessments and the change scores. As the raincloud plots of individual courses of performance suggest in Figure A3, there is a very high correlation among assessments of 0.95 or higher, in particular for adjacent assessment times: 0.97 across the first therapy period and 0.99 across the second therapy period. Interestingly, the initial performance level is not significantly correlated with the change in performance for the first period, the whole therapy phase, or the differential change in P2 versus P1. There only was a moderate size correlation of 0.59 (significantly different from zero at the 1% level) between the initial level of aphasic impairment and the change in performance across the period of combined treatment such that less impaired PwA benefited somewhat more from the combined treatment approach and more impaired PwA benefited somewhat less.

Age was not related to any of the assessments or to the change scores. Duration was moderately negatively correlated with the initial period change score (r = −0.54, *p* < 0.01) and somewhat less so with the change score across the whole treatment phase (r = −0.41, *p* < 0.05) and the differential change score (r = −0.2, *p* < 0.01), thus favoring PwA with a somewhat longer duration in the early phase postonset. For the BIAS group, no correlational analyses were performed because of the small sample size.

Finally, a comparison of correlation and intraclass correlation (ICC) between the three pairs of assessments for the AAT profile level—as shown in Table 4—demonstrates that the correlation coefficient is not the most adequate measure of retest reliability when there is a varying degree of change in the level of performance across assessment occasions. A one-factor repeated-measures ANOVA with two time points was carried out first in JASP to obtain the mean squares for factor time with two levels, for (random effects) factor PwA with *n* = 27 levels, and the residual mean squares, in which interaction among time and PwA (differences in steepness of slope) and additional random error are confounded.

## 4. Discussion

Our observational study has shown for the first time that NIBS stimulation protocols can be successfully implemented in an early rehabilitation routine, and this study provides the first evidence that an individually tailored anodal tDCS can improve the outcome of behavioral aphasia therapy in subacute aphasia. For the larger group of PwA assessed with the AAT, the improvement was significantly larger than what could be expected from spontaneous recovery, based on a historical control group.

It was also shown that tDCS as an adjuvant to aphasia therapy is well tolerated by PwA of different subtypes and severity levels in the acute stage and that side effects are perceived differently but in all cases are limited to a moderate level. 

Our individually tailored diagnostic and therapeutic procedure showed a significant add-on effect of behavioral SLT combined with an anodal tDCS over language-relevant areas of the left hemisphere on specific and overall language skills/functions compared to a preceding period of behavioral SLT alone. We have demonstrated this add-on for two samples of PwA using two different, severity-specific, established German language test batteries, the AAT and the BIAS-R. Although both samples improved in both therapy periods, P1 and P2, for both the AAT and the BIAS-R groups, the overall improvement in P2 for the primary outcome measures was significantly greater than in P1. The effect, however, was even more substantial in the AAT group.

### 4.1. tCDS in Combination with SLT in the Subacute Stage of Aphasia

While the positive effect of anodal tDCS in combination with SLT for patients with chronic aphasia has already been shown in many studies [14,16], the few studies applying tDCS together with SLT in persons with subacute aphasia report inconsistent results. Spielmann et al. [28] found in their randomized controlled study with 58 PwA no effect of an additive anodal 1 mA stimulation for 20 min over the left inferior frontal gyrus on a word-finding treatment compared to sham stimulation. A crucial difference between our study and Spielmann et al. [28] is that they had no information about the underlying brain lesions and consistently stimulated above the left IFG regardless of the lesion. It is possible that the stimulation was not applied over spared brain tissue in all patients. Stockbridge et al. [29] also found no significant effect of anodal tDCS versus sham stimulation on fifteen sessions of computer-delivered naming treatment on a naming task in 58 PwA (tDCS *n* = 30, sham *n* = 28). However, they did find a positive influence on functional communication. Stockbridge et al. used a hierarchical method to identify an optimal stimulation site. The first choice was the area of greatest activation, as revealed by fMRI, in the temporal lobe during a naming task. Since less than half of the participants agreed to this additional imaging, the effect of fMRI-guided stimulation location could not be evaluated. If an fMRI was refused, lesion-based localization was chosen. According to the hierarchical decision approach, an undamaged area of the left cortex was selected. Preference was given to stimulating the left IFG, followed by the left STG or the prefrontal cortex. The group also compared the effect of tDCS in patients with subacute and chronic aphasia and reported a greater effect on people with aphasia in the subacute stage. In our study, we followed a similar hierarchical theory-guided scheme. Since we did not have fMRI to measure task activity, we stimulated over preserved tissue of the left IFG after analyzing structural imaging. In the case of extensive lesions involving frontal and temporal areas, stimulation was applied over M1, and in the case of a scar after cranioplasty, the anode was placed over the FPC.

You and colleagues [30] compared the effect of SLT in combination with anodal tDCS over the left superior temporal gyrus (*n* = 7), cathodal tDCS over the right superior temporal gyrus (*n* = 7), and sham stimulation (*n* = 7) in individuals with subacute aphasia. After multimodal therapy that included both language comprehension and language production tasks, they observed, in addition to linguistic improvements in all groups, significant progress in auditory speech comprehension after cathodal stimulation relative to the other groups. However, You and colleagues discussed that in four out of seven participants in the anodal group, the stimulation may have been over necrotic tissue, while only one of the participants in the cathodal group had a lesion of the STG, which could have influenced the results. 

### 4.2. Primary Outcome Measures

Primary outcomes for both samples show the improvement in P2 with tDCS was significantly greater than in P1. The effect was even greater for the AAT sample in comparison to the BIAS-R sample.

One reason might be the initial severity of aphasia, which is reported as a highly relevant prognostic factor in many studies [67,68,69,70] and which was significantly higher in the BIAS-R sample than in the AAT sample. Although we did not detect an effect of initial severity on recovery within the two samples, we cannot rule out an influence of initial severity, which could account for the different development of the two samples. In addition, because the time postonset was longer in the BIAS-R sample, the influence of spontaneous remission was probably lower, which reduces the absolute improvement in P1 more than in P2 due to a fading influence of spontaneous recovery over the course of time. If spontaneous recovery data for the BIAS-R had been available to make the same correction for the BIAS-R sample, the difference between P2 and P1 might have been greater. However, it should be emphasized that the initial severity of the aphasia did not inhibit the add-on effect, but possibly only limited it. Thus, patients with severe aphasia can also benefit from additive tDCS. In accordance with the work of Wilson et al. [70], we found no effect of age, sex, or stroke type on recovery or responsiveness to tDCS in both groups.

An additional factor that could have favored the AAT sample is the relative sparing of the left Broca’s area or the surrounding tissue. In a recent longitudinal connectome study, Wilmskoetter et al. [71] identified regional controllability of the left inferior frontal gyrus as a strong predictor for positive treatment outcomes. Thus, close network connectivity in the left IFG appears to be an important cause of successful recovery. In contrast to 26/27 PwA in the AAT sample, only 5/10 PwA in the BIAS-R sample received stimulation over the left IFG (due to extended lesions or a scar after hemicraniectomy), which could have contributed to the difference in add-on effects between the two samples.

### 4.3. Secondary Outcome Measures

Within the AAT sample, we found significantly greater add-ons in P2 for overall severity (profile level) and all five subtests of the AAT as well as the average spontaneous speech score. These improvements are consistent with a network effect with activation of the residual left hemispheric language network, which supported the participants’ individually tailored but generally multimodal aphasia therapy.

The significant add-on by tDCS on spontaneous speech was not initially expected, as many of tDCS studies with PwA only find an improvement for trained items in trained tasks [16]. However, there are examples of generalization or transfer effects. After bilateral stimulation, Marangolo et al. [72] found not only improvements for trained items but also improvements for untrained items and tasks such as naming and picture description, which were not part of the SLT regimen (repetition of words and sentences). Meinzer et al. [21] found transfer effects from a computerized naming treatment on functional communication after real anodal stimulation over M1. Stockbridge et al. [29] detected no effect on naming after unilateral, left hemispheric stimulation in contrast to sham stimulation but positive effects on picture description. It should be noted that the evaluation of the picture description is generally based on different criteria than the analysis of spontaneous speech in the AAT. While the picture description is primarily evaluated according to content and efficiency [73,74,75], i.e., mainly semantic criteria, the evaluation of spontaneous speech in the AAT is also based on semantic properties (communication, automated speech, semantics) in three of the six subscales, while the remaining three scales (articulation, phonology, and syntax) are evaluated on formal linguistic criteria. Based on our analysis, we were unable to determine which and how many of the subscales drove the effect in P2 due to the limited degree of differentiation of the six-point rating scales employed.

The significant add-on effect on BIAS-R mean percent correct can also best be explained by a network effect that leads to a slight, more homogenous improvement in various tasks via increased activation. The lack of a significant add-on effect of tDCS on specific subtests, except for repetition and naming, could be due to the stronger and longer-lasting language impairments in all, but especially in the expressive, modalities. It is quite reasonable to assume that the treatment time between the assessments was too short for these severely affected patients to achieve significant specific improvements—except for the subtests, which are supported by the use of cues in the BIAS-R and are usually easier to evoke responses even in severe aphasia. As already discussed for the primary outcome measures, the possibility of a correction for spontaneous remission might have also increased the difference between P2 and P1 for the secondary outcomes in the BIAS-R sample.

In order to obtain the patients’ individual perception of the success of the therapy, it would have been useful to systematically record this perspective, as treatment success cannot be determined by changes on standardized outcome measurement instruments alone. This perspective is assessed by the minimal important changes (MIC) criterion, which quantifies clinically significant changes. In German, there are no standardized MIC values for the early phase of aphasia [76], and other patient-reported outcome measures (PROMs) are only gradually becoming established, like the SAQOL-39 [4,77], which is currently being translated into German. Nevertheless, a simple Likert scale supported by visual symbols and understandable for people with aphasia could have been used, at least for informal recording of the perceived success of the therapy [76].

To successfully implement NIBS protocols in clinical routine, good tolerability and high acceptance by PwA are required, which is why we recorded the discomfort after each session. The level of discomfort/pain caused by the stimulation across all sessions was rated as mild (<2 on a scale of 0–10) for all three stimulation sites on average. Nevertheless, there were individual differences. Two of the thirty-one PwA with stimulation over the IFG rated the discomfort as above 6, fourteen of thirty-one PwA rated <1. The rating remained largely stable over the 10 sessions. Habituation effects or increases in discomfort were not observed. Although the stimulation was well tolerated by all PwA, a routine recording of side effects should always be performed to involve the PwA in the treatment, document changes, and detect potential differences between the sensitivity of different stimulation sites.

### 4.4. Use of Different Severity-Specific Language Tests

We could show that the therapy outcomes in both phases could be demonstrated most sensitively and differentiated with the AAT. We chose the BIAS-R as an alternative diagnostic instrument for participants with severe expressive difficulties to avoid floor effects and missing values in the expressive subtests in the AAT. The BIAS-R allows the use of semantic and/or phonological cues in several expressive tasks to facilitate word retrieval. In addition, the BIAS-R also includes tasks that require the recall of non-propositional language, such as serial speech or counting, which might engage right hemisphere language areas [78], which are especially activated in the early subacute phase [79,80]. Another argument in favor of maintaining the severity-specific test procedures is the limited capacity and frustration tolerance of participants with severe aphasia. The BIAS-R can usually be finished within one session in about 40 min (1–2 sessions), while the assessment with the AAT normally lasts between 60 and 90 min. The cues in the BIAS-R also provide the SLT with direct information for therapy planning and the final “successful” recall counteracts the frustration of severely affected patients.

### 4.5. Stimulation Site

Anodal tDCS over the left IFG has been shown to primarily recruit left perilesional regions, structures that are associated with the best recovery from aphasia [16,26,81,82,83], and therefore this stimulation site is addressed in most studies [14,26]. However, this presupposes that perilesional, reactivatable tissue is spared, which was not controlled in all studies. Based on the available evidence, the left IFG was chosen as the stimulation site in our study if reactivatable tissue was present in this area. Most of our participants received stimulation over the left IFG (26/27 participants in the AAT sample and 5/10 in the BIAS-R sample). In participants (*n* = 3) with extensive lesions in the area of the IFG (and the STG), the anode was placed over M1 with the goal to activate the whole distributed language network via its connections to the primary motor cortex [50,52]. The positive effect of anodal stimulation via M1 on the outcome of aphasia therapy is being reported in an increasing number of studies [21,51] and is currently being investigated in a multicenter, Germany-wide randomized controlled trial [84]. 

This network perspective and approach for describing and modeling healthy and aphasic language have become established in recent years. As shown in many (lesion) studies [18,85,86], Broca’s and Wernicke’s areas are important nodes for language processing, but the connections between them are just as important. And just as aphasia can also result from damage to the connecting pathways [87,88], these pathways can also be used as a gateway into the language network. The physiological effects of tDCS on remote areas and the effect of tDCS on functional connectivity have been described in several studies [89,90,91]. With regard to language, Darkow et al. [92] were able to show that 20 min of 1 mA excitatory tDCS over the motor cortex, in contrast to sham stimulation, increased activity in the residual language network of PwA. The effect of increased connectivity in the left hemisphere was also found by Marangolo et al. [72]. They showed that bilateral tDCS (anodal left and cathodal right) in contrast to sham stimulation in individuals with chronic aphasia not only improved trained tasks but also had transfer effects on non-trained tasks and connectivity in the left hemisphere. Patients in the real stimulation group exhibited the greatest recovery not only in terms of better accuracy in articulating the treated stimuli but also for untreated items from different subtests of the administered language test.

The increasing contribution of domain-general networks (including, among others, the left FPC) after the interruption of more specific functions to the reorganization process after brain damage—such as stroke—was demonstrated by Hartwigsen and Volz [88] and Dreyer et al. [93], who showed that increased activity in the default mode network was associated with higher gains in AAT performance after intensive SLT. For the early phase of aphasia recovery, Stockert et al. [79] also demonstrated the special contribution and activation of domain-general networks. From this connection and interaction between default networks and language network, the three participants with the anode positioned over the left FPC could have benefited, and this more generalized stimulation of the language network could also offer a plausible explanation for an improvement in various linguistic modalities. 

There are now several tDCS studies that have investigated individualized protocols—e.g., identifying individual stimulation sites through a prior fMRI examination to determine responsive brain areas in various linguistic tasks—and yielded differentiated findings [25,40,94,95]. In some studies, rather than the usual 5 × 7 cm electrodes, so-called high-definition tDCS electrodes (HD-tDCS), which have a higher focal resolution, have been used [94,96].

As discussed by Shah-Basak et al. [25], functional imaging is certainly the best way to identify spared and functionally active tissue and is a reasonable approach for research and specialized research labs. However, it is difficult to implement in everyday clinical practice at a rehabilitation clinic [25]. Zumbansen et al. [97] suggest a combination of behavioral and imaging criteria to identify suitable participants and stimulation sites for NIBS treatments. In our study, we relied on CT/MRI imaging to ensure that there was spared tissue under the targeted stimulation site and the results suggest that structural imaging provides sufficient information to identify a suitable target area. The procedure may also differ between the application of tDCS in the chronic and in the subacute phase. In the early stage of aphasia, the specific stimulation site might not be as crucial as in the chronic phase, as long as it is possible to involve the remaining left hemispheric network (if spared) in language processing through stimulation and appropriate paired language therapy [25].

### 4.6. Implementation in Clinical Practice

Our aim was to implement the application of tDCS in combination with language therapy—for which there is growing evidence but which still raises unanswered questions—in a real clinical setting with an extended patient group with subacute aphasia and to evaluate the outcome of the stimulation on the increase in linguistic performance of PwA. We did not intend to additionally accompany and interpret this implementation systematically within a theoretical framework, as the “hybrid approaches” of the implementation sciences do [35,98,99,100]. Nevertheless, we can subsequently identify and name barriers and facilitators. In the case of implementing NIBS protocols in clinical practice, there is a particularity in the field of non-invasive brain stimulation. Although there are (some) established procedures and recommendations from RCTs for a number of selected patient groups in chronic aphasia [14,16,26,101], the exact physiological effects of brain stimulation are not yet sufficiently known, even in these groups. Above all, it is unclear whether the results found in studies on relatively homogeneous groups can also be transferred to heterogeneous samples in clinical practice. From an implementation science perspective, barriers to adopting NIBS protocols from the chronic phase in a straightforward way are structural and institutional givens like limited personal resources, lack of high-quality imaging, and—more importantly—barriers inherent to the patients. In a neurology ward, the physiological conditions of aphasic patients and the clinical manifestations as well as their needs are very heterogeneous, which constrains a strictly standardized, methodologically more sound approach. The limited resilience of patients in the early poststroke phase also restricted the number of assessments administered. In contrast to specialized research labs and university contexts, clinical neurological rehabilitation facilities financed by health insurance companies do not have the personnel capacity for research and must guarantee a high amount of therapy and good nursing and medical care. For these reasons, for example, we were unable to apply a consistent sham stimulation in P1, which would have resulted in blinding of the PwA.

However, there were also numerous facilitators in terms of implementation science. Some of the therapists and physicians already had theoretical and practical knowledge of the NIBS method used here and tDCS, and all other colleagues were willing to acquire this knowledge and apply it in practice through training courses at the clinic. Adherence to the planned study design and therapies was supported by prioritization, in case of therapist illness, and adherence to the study design and therapy regimen was guaranteed through a back-up system among the SLTs. The high motivation of the patients and their relatives as well as the consistent acceptance of the study protocol and the stimulation were also particular facilitators. Apart from the three early discharges from the clinic, no participant interrupted treatment—neither in the first nor in the second period—so that all data sets could be analyzed. 

### 4.7. Limitations

First, we had no “real” control group or sham stimulation. We implemented no blinding of participants, SLPs, and investigator, and therefore the positive expectation or bias of the participants could have influenced the results. Blinding of the PwA would have been possible through sham stimulation during the first therapy phase, but this could not be implemented in everyday clinical practice. However, the possible placebo effect may have been compensated for by the participants’ concerns about the side effects of electrical stimulation. During the detailed explanatory discussions, participants and their relatives repeatedly expressed concerns about the possible side effects of electrical stimulation on such a sensitive area of the body as the brain.

Period 1, consisting of exclusive SLT, may be considered an intraindividual control condition for judging the efficacy of the add-on tDCS stimulation. The sequence P1/P2 was, however, identical for all PwA, and the findings for P2 might also in part reflect a cumulative effect of 20 therapy sessions over both treatment periods. A cross-over design would perhaps have been methodologically appropriate, but we had deliberately chosen this sequence to keep the influence of spontaneous remission in P2 lower than in P1. A further argument in favor of maintaining the sequence of periods is the so-called “after effect”. These are persisting effects of tDCS far beyond the stimulation period [102,103,104], which presumably influence both local activity and network activation. In studies with a cross-over design and people with chronic aphasia, attempts are made to neutralize this effect with a “wash-out period” lasting days or weeks, which is inserted between the treatment periods [22,72,105,106]. A consistent implementation of a cross-over design in the subacute phase would have required, in addition to the implementation of a wash-out period, an additional assessment after the wash-out period and prior to P2 in order to record the changes in the wash-out period (after effects and spontaneous remission) between the end of P1 and the start of P2. However, this would have prolonged our protocol beyond the normal rehabilitation period and, above all, it would have further increased the difference between the influence of spontaneous recovery between P1 and P2. Furthermore, such a prolongation would have probably led to conflicts with the insurance companies and to more study drop-outs due to patient discharges.

A limitation—but also a strength—of our approach was that the participants’ therapy focused individually on different combinations of impaired language modalities and was better able to incorporate the individual wishes and goals of the participants than, for example, a training protocol with controlled but less relevant items for everyday life. However, as all participants had impairments in several modalities (see Table A4), we applied a multimodal approach that had been shown to be effective in the subacute phase, individually tailored to different levels of severity and complexity. Since the method of therapy was maintained during P1 and P2 and was only adapted to the linguistic performance of the participants, this factor should have had little influence on the degree of the improvements in P1 and P2.

Further limitations arising from the clinical procedure were, for example, that not all patients in the clinic were systematically screened for inclusion in the study, and this could have resulted in a possible selection bias. Furthermore, the repetition of the same assessment within a relatively short period of time could have led to a learning effect. However, as no feedback was given regarding the participants’ reactions in either test procedure, this is rather unlikely. As most of the participants did not stay in the clinic long enough and had been discharged to their homes throughout Germany, we were not able to conduct a follow-up.

Additional assessments with patient-reported outcome measures (PROMs) and cognitive functions such as attention, memory, or executive function would also be desirable. To minimize the test burden on our participants and in the absence of suitable test procedures for recording PROMs, we limited the number of tests. In future studies, at least a screening of cognitive functions and an assessment with a PROM that is easy to understand for PwA should be implemented to record possible differential therapy effects (e.g., cognitive vs. linguistic), particularly in the case of stimulation at different stimulation sites (e.g., FPC vs. IFG).

## 5. Conclusions

The present study showed for the first time that individual tDCS protocols as an adjuvant to behavioral aphasia therapy can be successfully and safely implemented in the daily routine of an early rehabilitation clinic. The results demonstrate that add-on anodal tDCS over spared left hemispheric areas can result in significantly larger increases in language performance than behavioral aphasia therapy alone in individuals with subacute aphasia of different types, severity, and duration, who benefit to a similar degree from a methodologically controlled but clinically feasible approach.

## Figures and Tables

**Table 1:** Patient characteristics.

Group		AAT	BIAS-R
Etiology (stroke)	IS	23	7
	IHS	4	3
Stimulation site (left)	IFG	26	5
	M1		3
	FPC	1	2
Sex	Male	19	5
	Female	8	5
Age (years)	M (SD)	61.96 (11.63)	58.3 (11.57)
	Median (range)	63 (36–83)	56 (38–80)
Education (years)	M (SD)	14.11 (2.91)	14.6 (3.31)
	Median (range)	16 (9–18)	14.5 (10–20)
Duration (days)	M (SD)	49.33 (28.04)	76.6 (40.79)
	Median (range)	46 (13–107)	68 (33–145)
Aphasia type ^1^	Global	6	
	Wernicke	10	
	Broca	6	
	Anomic	5	
	Subacute aphasia		10
Aphasia severity ^2,3^	Severe	10	10
	Moderate	10	
	Mild	6	
	Residual	1	
Severity ERBI ^4^ (−325–105)	M (SD)	+17.59 (31.72)	−57 (42.44)
	Median (range)	+25 (−65 to +60)	−65 (−125 to +40)
Severity FIM ^5^ (18–126)	M (SD)	75.56 (20.58)	56.6 (15.86)
	Median (range)	81 (29–107)	54.5 (38–90)

IS = Ischemic; IHS = Intracerebral hemorrhagic; IFG = Inferior frontal gyrus; M1 = Motor cortex; FPC = Frontopolar cortex; F = Female; M = Male; ^1^ Aphasia diagnosis according to Aachen Aphasia Test (AAT)/BIAS-R does not classify into aphasia types (subacute aphasia). ^2^ Severity according to AAT profile level value (T-score); severe < 46, moderate 46–54.9, mild 55–62.9, residual ≥ 63. ^3^ Severity according to BIAS-R percentile rank (PR); severe < 23, moderate 24–59, mild 62–88, residual ≥ 89. ^4^ Early Rehabilitation Barthel Index (ERBI), at beginning of rehabilitation stay. ^5^ Functional Independence Measure (FIM), at beginning of rehabilitation stay.

**Table 3:** Analysis of difference scores between assessments: one-sided *t*-tests for improvement beyond the expected improvement due to spontaneous recovery (see Table A1A in Appendix A) and differentially stronger improvement for period 2 compared to period 1 (taking again account of the difference in spontaneous recovery for each therapy period), as well as effect size estimates (Cohen’s d with 95% one-sided confidence interval) and Bayes factor (BF) for the degree of evidence for improvement (BF_+0_) or for lack of improvement (BF_0+_). A: AAT profile level T-score, average spontaneous speech rating, and subtest raw scores; B: BIAS-R average percentage value T-score and subtest percentage values, tested against zero improvement, since no information on spontaneous recovery is available for the BIAS-R.

**A**	**Period 1**			**Period 2**			**Period 2 − 1**			**Period 1 + 2**		
**AAT (*n* = 27)**	t(26) * **(p-v.)**	**Cohen d** **[95%-CI]**	**Bayes** **Factor**	t(26) * **(p-v.)**	**Cohen d** **[95%-CI]**	**Bayes** **Factor**	t(26) * **(p-v.)**	**Cohen d** **[95%-CI]**	**Bayes** **Factor**	t(26) * **(p-v.)**	**Cohen d** **[95%-CI]**	**Bayes** **Factor**
Profile	0.25 (0.40)	0.19 [−0.27–∞]	BF_0+_ 4.01	**7.94** (<0.001)	1.53 [1.05–∞]	BF_+0_ 1.3 × 10^8^	**6.49** (<0.001)	1.25 [0.82–∞]	BF_+0_ 48,879	**4.01** (<0.001)	0.77 [0.40–∞]	BF_+0_ 136
TT	1.69 (0.052)	0.32 [−0.01–∞]	BF_0+_ 1.33	**4.63** (<0.001)	0.89 [0.51–∞]	BF_+0_ 585	1.39 (0.089)	0.27 [−0.06–∞]	BF_0+_ 1.16	**4.44** (<0.001)	0.85 [0.48–∞]	BF_+0_ 58
REP	0.80 (0.21)	0.16 [−0.17–∞]	BF_0+_ 2.36	**4.51** (<0.001)	0.87 [0.49–∞]	BF_+0_ 438	**3.49** (<0.001)	0.67 [0.32–∞]	BF_+0_ 42	**3.08** (0.002)	0.59 [0.24–∞]	BF_+0_ 17
WRIT	0.76 (0.23)	0.15 [−0.17–∞]	BF_0+_ 2.47	**2.83** (0.004)	0.54 [0.20–∞]	BF_+0_ 10.2	1.06 (0.15)	0.21 [−0.12–∞]	BF_0+_ 1.75	**3.19** (0.002)	0.61 [0.26–∞]	BF_+0_ 22
NAM	0.28 (0.39)	0.05 [−0.26–∞]	BF_0+_ 3.91	**4.17** (<0.001)	0.80 [0.43–∞]	BF_+0_ 196	2.17 (0.019)	0.42 [0.08–∞]	BF_+0_ 2.96	**3.19** (0.002)	0.62 [0.26–∞]	BF_+0_ 7.55
COMP	−0.76 (0.77)	−0.15 [−0.46–∞]	BF_0+_ 7.99	**3.84** (<0.001)	0.74 [0.37–∞]	BF_+0_ 91	**2.98** (0.003)	0.57 [0.23–∞]	BF_+0_ 14	**3.28** (0.001)	0.63 [0.28–∞]	BF_+0_ 27
**B**	**Period 1**			**Period 2**			**Period 2−1**			**Period 1 + 2**		
**BIAS (*n* = 10)**	t(9) * **(p-v.)**	**Cohen d** **[95%-CI]**	**Bayes** **Factor**	t(9) * **(p-v.)**	**Cohen d** **[95%-CI]**	**Bayes** **Factor**	t(9) * **(p-v.)**	**Cohen d** **[95%-CI]**	**Bayes** **Factor**	t(9) * **(p-v.)**	**Cohen d** **[95%-CI]**	**Bayes** **Factor**
Profile	**4.06** (0.001)	1.29 [0.55–∞]	BF_+0_ 34	**6.80** (<0.001)	2.15 [1.15–∞]	BF_+0_ 700	2.22 (0.027)	0.70 [0.10–∞]	BF_+0_ 3.20	**6.26** (<0.001)	1.98 [1.03–∞]	BF_+0_ 410
ASV	1.69 (0.063)	0.54 [−0.04–∞]	BF_+0_ 1.65	**3.68** (0.003)	1.16 [0.46–∞]	BF_+0_ 21	0.31 (0.38)	0.10 [−0.43–∞]	BF_0+_ 2.56	**3.52** (0.003)	1.11 [0.42–∞]	BF_+0_ 17
AUT	**3.26** (0.005)	10.03 [0.36–∞]	BF_+0_ 12	**5.46** (<0.001)	1.73 [0.50–∞]	BF_+0_ 176	1.37 (0.102)	0.43 [−0.12–∞]	BF_+0_ 1.14	**6.21** (<0.001)	1.96 [1.02–∞]	BF_+0_ 390
ELIZ	0.23 (0.41)	0.07 [−0.45–∞]	BF_0+_ 2.71	**2.49** (0.017)	0.79 [0.11–∞]	BF_+0_ 4.59	1.71 (0.060)	0.54 [−0.0–∞]	BF_+0_ 1.70	**2.04** (0.036)	0.65 [0.05–∞]	BF_+0_ 2.56
NACH (*n* = 9)	**4.14** (0.002)	10.38 [0.57–∞]	BF_+0_ 31	**3.09** (0.007)	1.03 [0.32–∞]	BF_+0_ 9.19	1.94 (0.044)	0.65 [0.02–∞	BF_+0_ 2.91	**4.34** (0.001)	1.45 [0.62–∞]	BF_+0_ 39
LSV	**2.84** (0.010)	0.90 [0.26–∞]	BF_+0_ 7.23	**2.75** (0.011)	0.87 [0.23–∞]	BF_+0_ 6.40	0.14 (0.45)	0.04 [−0.48–∞]	BF_0+_ 3.36	**4.63** (<0.001)	1.46 [0.67–∞]	BF_+0_ 68
LLES	**3.36** (0.004)	10.06 [0.38–∞]	BF_+0_ 14	1.61 (0.072)	0.51 [−0.06–∞]	BF_+0_ 1.49	−0.05 (0.52)	−0.02 [−0.54–∞]	BF_0+_ 2.56	**2.78** (0.011)	0.88 [0.24–∞]	BF_+0_ 6.65
SCHR	1.38 (0.100)	0.44 [−0.12–∞]	BF_+0_ 1.15	**2.81** (0.010)	0.89 [0.25–∞]	BF_+0_ 6.92	0.35 (0.37)	0.11 [−0.42–∞]	BF_0+_ 2.47	**3.30** (0.005)	1.04 [0.37–∞]	BF_+0_ 13

* In bold font: significant also after Holm correction for family of five tests (AAT) and seven tests (BIAS-R).

**Table 4:** Intraclass correlations (ICC) for consistency and stability (agreement) of AAT performance over the therapy periods.

AAT Profile Level	Period		
	1	2	1 + 2
MS (time) ^#^	27.879	126.745	273.510
MS (PwA) ^#^	103.846	127.602	117.610
MS (residual) ^#^	1.718	0.940	4.035
Estim. σ^2^ (time)	0.969	4.659	9.981
Estim. σ^2^ (PwA)	51.064	63.331	56.7875
Estim. σ^2^ (residual)	1.718	0.940	4.035
Pearson r	0.970	0.993	0.951
ICC (consistency) ^§^	0.967	0.985	0.934
ICC (agreement) ^§^	0.950	0.919	0.802

^#^ From a repeated-measures one-factorial ANOVA with 2-level factor “time”, MS: mean squares, Estim. σ^2^: estimated variance component, ^§^ ICC(consistency) = ICC(3,1), ICC(agreement) = ICC(2,1), as defined by Shrout and Fleiss [66].

**Figure A8:**
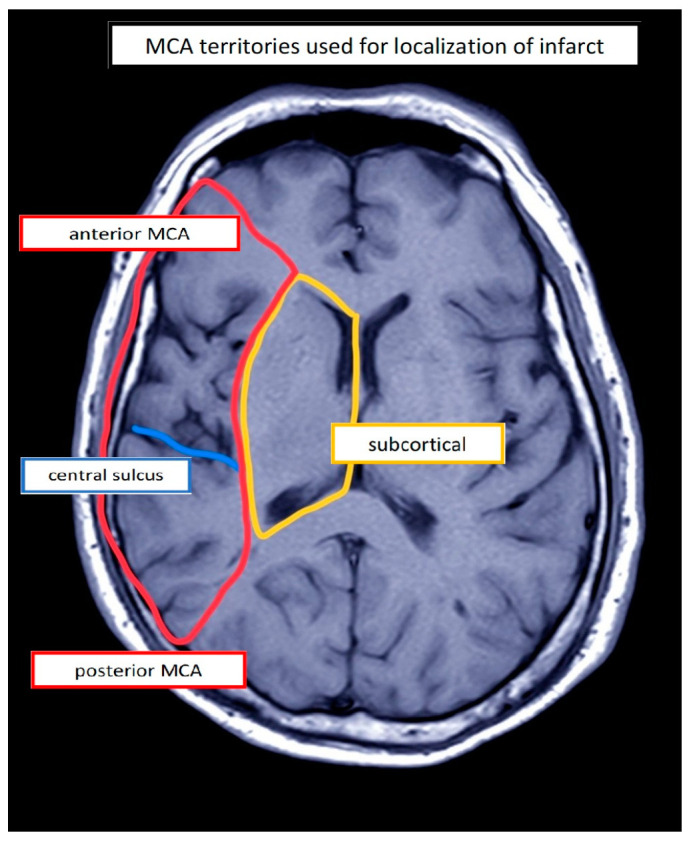
Lesion location of infarct—MCA territories.

**Figure A9:**
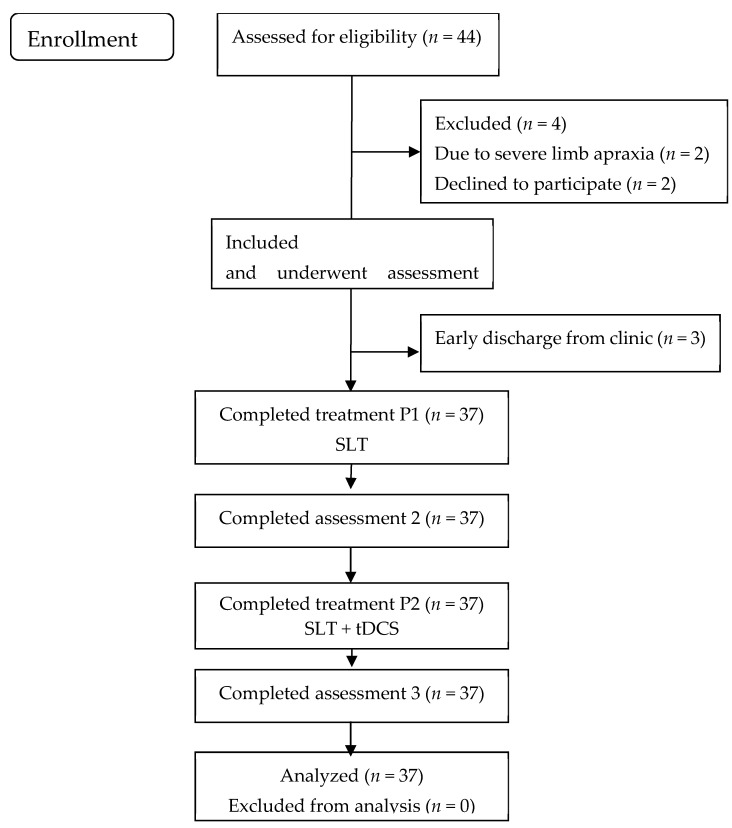
Participant flow diagram.

## Data Availability

The original contributions presented in the study are included in the article and Appendix A, Appendix B, Appendix C, Appendix D and Appendix E, further inquiries can be directed to the corresponding author/s.

## Abbreviations

AAT	Aachen Aphasia Test
BIAS-R	Bielefeld Aphasia Screening-Reha
FIM	Functional Independence Measure
FPC	frontopolar cortex
IFG	inferior frontal gyrus
M1	primary motor cortex
MIC	minimal important changes
PwA	person with aphasia
PROM	patient-reported outcome measure
SLT	speech and language therapy
SLP	speech and language pathologist
tDCS	transcranial direct current stimulation
(r)TMS	repetitive transcranial magnetic stimulation

## Appendix A. Additional Tables

**Table A1:** Descriptive information (arithmetic mean (M), standard deviation (SD)) on performances across the therapy period; A: AAT profile level T-score, average spontaneous speech rating, and subtest raw scores; B: BIAS-R average percentage value T-score and subtest percentage values.

**A**	**Assessment**			**Difference**					
**AAT**	**T1**	**T2**	**T3**	**Period 1**	**Period 2**	**Period 2 − 1**	**Period 1 + 2**	**Expected**	
(*n* = 27)	M (SD)	M (SD)	M (SD)	M (SD)	M (SD)	M (SD)	M (SD)	P1	P2
Profile level	49.60 (7.02)	51.04 (7.50)	54.10 (8.50)	1.43 (81.86)	3.06 (1.37)	1.63 (1.61)	4.50 (2.84)	1.34	0.97
Spont.	2.83 (1.91)	2.95 (0.82)	3.27 (0.82)	0.12 (0.47)	0.32 (0.29)	0.19 (0.56)	0.44 (0.54)	-	-
TT	27.78 (16.10)	24.04 (16.24)	19.00 (15.67)	−3.74 (6.17)	−5.04 (4.23)	−1.30 (6.63)	−8.78 (8.24)	1.74	1.27
REP	102.70 (37.80)	107.82 (33.68)	117.82 (28.77)	5.11 (8.61)	10.00 (8.37)	4.89 (8.82)	15.11 (14.51)	3.78	2.74
WRIT	39.44 (26.68)	44.34 (26.76)	50.56 (26.85)	4.93 (7.37)	6.19 (6.36)	1.26 (11.62)	11.11 (7.38)	3.85	2.73
NAM	51.74 (35.04)	56.70 (36.71)	67.15 (35.39)	4.96 (11.57)	10.44 (9.33)	5.48 (16.38)	15.41 (13.17)	4.34	2.97
COMP	77.33 (18.30)	79.59 (19.66)	89.11 (17.44)	2.26 (7.37)	9.52 (9.62)	7.26 (14.24)	11.78 (9.53)	3.34	2.42
**B**	**Assessment**			**Difference**					
**BIAS**	**T1**	**T2**	**T3**	**Period 1**	**Period 2**	**Period 2 − 1**	**Period 1 + 2**		
(*n* = 10)	M (SD)	M (SD)	M (SD)	M (SD)	M (SD)	M (SD)	M (SD)		
Profile level	37.40 (3.81)	39.35 (2.97)	42.35 (2.45)	1.95 (1.52)	3.00 (1.39)	1.05 (1.50)	4.95 (2.50)		
ASV	63.91 (12.57)	69.33 (11.26)	75.95 (9.35)	5.42 (3.60)	6.62 (5.70)	1.20 (12.40)	12.04 (10.82)		
AUT	42.98 (15.98)	52.22 (18.39)	66.90 (17.86)	9.25 (8.96)	14.68 (8.51)	5.44 (12.53)	23.93 (12.18)		
ELIZ	40.80 (6.94)	41.37 (7.11)	48.31 (6.31)	0.57 (7.72)	6.94 (8.80)	6.37 (11.76)	7.51 (11.65)		
NACH (*n* = 9)	28.88 (25.39)	36.30 (26.12)	61.09 (23.57)	7.42 (5.38)	24.79 (24.05)	17.36 (26.79)	32.21 (22.28)		
LSV	58.49 (18.94)	63.79 (20.00)	69.54 (19.59)	5.30 (5.90)	5.75 (6.62)	0.45 (10.00)	11.05 (7.55)		
LLES	24.97 (26.14)	33.28 (24.01)	41.32 (27.51)	8.31 (7.83)	8.04 (15.85)	−0.27 (16.68)	16.35 (18.61)		
SCHR	8.33 (12.35)	16.60 (19.80)	27.73 (22.83)	8.27 (18.94)	11.13 (12.53	2.86 (26.20)	19.40 (18.58)		

**Table A2:** AAT performance differences tested against zero for profile level T-score, average spontaneous speech rating, and subtest raw scores.

AAT	Period 1			Period 2			Period 2 − 1			Period 1 + 2		
(*n* = 27)	t(26) * (p-v.)	Cohen d [95%-CI]	Bayes Factor	t(26) * (p-v.)	Cohen d [95%-CI]	Bayes Factor	t(26) * (p-v.)	Cohen d [95%-CI]	Bayes Factor	t(26) * (p-v.)	Cohen d [95%-CI]	Bayes Factor
Profile	**3.99** (<0.001)	0.77 [0.40–∞]	BF_+0_ 131	**11.61** (<0.001)	2.24 [1.63–∞]	BF_+0_ 2 × 10^9^	**5.29** (<0.001)	1.02 [0.62–∞]	BF_+0_ 2840	**8.23** (<0.001)	1.58 [1.10–∞]	BF_+0_ 2.5 × 10^6^
Spont.	1.38 (0.089)	0.27 [−0.06–∞]	BF_0+_ 1.16	**5.64** (<0.001)	1.09 [0.68–∞]	BF_+0_ 6617	1.79 (0.043)	0.34 [0.02–∞]	BF_+0_ 1.56	**4.24** (<0.001)	0.82 [0.44–∞]	BF_+0_ 232
TT	**3.15** (0.002)	0.61 [0.26–∞]	BF_+0_ 20	**6.19** (<0.001)	1.19 [0.77–∞]	BF_+0_ 24,259	1.02 (0.16)	0.20 [−0.13–∞]	BF_0+_ 1.85	**5.53** (<0.001)	1.07 [0.66–∞]	BF_+0_ 5094
REP	**3.09** (0.002)	0.59 [0.25–∞]	BF_+0_ 17	**6.21** (<0.001)	1.20 [0.77–∞]	BF_+0_ 25,311	**2.88** (0.004)	0.55 [0.21–∞]	BF_+0_ 11	**5.41** (<0.001)	1.04 [0.64–∞]	BF_+0_ 232
WRIT	**3.47** (<0.001)	0.67 [0.31–∞]	BF_+0_ 40	**5.06** (<0.001)	0.97 [0.58–∞]	BF_+0_ 1629	0.56 (0.29)	0.11 [−0.21–∞]	BF_0+_ 3.02	**7.82** (<0.001)	1.51 [1.03–∞]	BF_+0_ 232
NAM	2.23 (0.017)	0.43 [0.09–∞]	BF_+0_ 3.27	**5.82** (<0.001)	1.12 [0.71–∞]	BF_+0_ 10,118	1.74 (0.047)	0.34 [0.01–∞]	BF_+0_ 1.45	**6.08** (<0.001)	1.17 [0.75–∞]	BF_+0_ 232
COMP	1.59 (0.062)	0.31 [−0.02–∞]	BF_+0_ 1.16	**5.14** (<0.001)	0.99 [0.59–∞]	BF_+0_ 2000	**2.65** (0.007)	0.51 [0.17–∞]	BF_+0_ 7.15	**6.42** (<0.001)	1.24 [0.80–∞]	BF_+0_ 232

* In bold font significant also after Holm correction for family of seven tests.

## Appendix C. Details of the Expected Spontaneous Recovery Estimation Procedure for AAT Performances

Calculation of the expected changes in AAT subtest performance over the two 2-week treatment periods is based on historical data from a multicenter spontaneous recovery study for German PwA [43]. The sample comprised global (*n* = 21), Wernicke (*n* = 19), Broca (*n* = 12), anomic (*n* = 32), and non-classifiable (*n* = 12) PwA examined 1, 4, and 7 months postonset. The average (rounded) raw score increases in AAT subtest performance were reported in Luzzatti et al. ([44]; Table 10b) and in Peitz et al. ([38]; Supplementary Table S1) with the expected change profile level T-score added with D_12_ or D_23_ labeling the differences between month 1 and month 4 or between month 4 and 7.

Since the time intervals between AAT assessments were longer in the spontaneous recovery study (3 months) than in the actual study (two consecutive two-week periods), we followed the approach developed by Peitz and coworkers to compute the expected change for intervals within the range of month 1 to month 7 per aphasic syndrome. They interpolated the course of spontaneous recovery with a non-linear, steeper course from 1–4 months postonset and a linear course from 4–7 months postonset. According to this interpolated course, we used multipliers smaller than (or equal to) 1 to calculate the raw score differences of spontaneous recovery for the shorter time intervals of the overall four weeks in our study (see Supplementary Table S2, in Peitz et al. [38]), which after rounding of the days postonset registered per PwA all fell in intervals for the early subacute group from that (Supplementary Table S2). Thus, the weight was 0.4 for D_12_ applied to PwA treated from month 1 to month 2, the weight was 0.35 when treated from month 2 to month 3, and the weight was 0.25 when treated from month 3 to month 4. Since the overall treatment phase of four weeks was furthermore split in two periods of two weeks each, the three weights applied to D_12_ for individual PwA were split as follows: 0.4 = 0.24 + 0.16, 0.35 = 0.2 + 0.15, and 0.25 = 0.13 + 0.12.

These corrections for individual PwA were then employed to compute the expected average spontaneous recovery score for the sample of *n* = 27 PwA, with thirteen persons falling in the month 1 to month 2 category, eight PwA in the month 2 to month 3 category, and six in the month 3 to month 4 category. In the final step, the frequency of standard aphasic syndromes per subsample was determined. This subsample information was then employed to compute weighted average of the individual expected change scores separately for both periods and each AAT subtest as well as the profile level. These weighted averages are listed in the two rightmost columns of Table A1.

## Appendix D. Additional Tables and Material

**Table A3:** Participant characteristics, individual data.

PwA	Etiology	Lesion Location ^1^	Stimulation Site (Left)	Sex	Age (Years)	Education (Years)	Duration (Days)	Assessment	Aphasia ^2^	Aphasia Severity ^3,4^	Initial Severity (ERBI) ^5^ (FIM) ^6^ (−325 to +105) (18–126)	
1	IS	a/sc	IFG	M	58	18	25	AAT	Wernicke	Moderate **^2^**	55	102
2	IS	a	IFG	F	82	10	33	AAT	Wernicke	Mild	40	62
3	IS	a	IFG	M	70	16	21	AAT	Wernicke	Severe	25	68
4	IS	sc	IFG	M	66	17	57	AAT	Anomic	Mild	60	86
5	IHS	sc	IFG	F	63	16	29	AAT	Anomic	Mild	−15	47
6	IHS	p/sc	IFG	F	57	17	25	AAT	Anomic	Residual	40	107
7	IS	a/p	IFG	M	69	16	107	AAT	Wernicke	Moderate	50	79
8	IHS	a/sc	FPC	M	41	16	77	AAT	Anomic	Moderate	−15	81
9	IHS	p	IFG	M	64	13	47	AAT	Wernicke	Moderate	−65	70
10	IS	a	IFG	F	64	13	13	AAT	Global	Severe	5	107
11	IS	a/sc	IFG	M	52	17	32	AAT	Global	Severe	35	92
12	IS	sc	IFG	M	36	12	29	AAT	Global	Severe	45	89
13	IS	a/sc	IFG	M	71	13	55	AAT	Broca	Mild	−30	53
14	IS	a/sc	IFG	M	48	17	15	AAT	Broca	Severe	60	84
15	IS	a/p	IFG	M	73	17	16	AAT	Wernicke	Severe	10	93
16	IS	sc	IFG	F	62	17	46	AAT	Global	Moderate	−20	52
17	IS	sc	IFG	M	50	17	99	AAT	Broca	Mild	10	80
18	IS	sc	IFG	M	58	10	83	AAT	Global	Severe	30	46
19	IS	a	IFG	F	67	10	56	AAT	Anomic	Mild	15	60
20	IS	a/p	IFG	M	66	10	64	AAT	Global	Severe	−10	47
21	IS	a	IFG	M	69	13	102	AAT	Broca	Moderate	−15	29
22	IS	p	IFG	F	81	10	66	AAT	Wernicke	Severe	25	92
23	IS	a	IFG	M	50	16	41	AAT	Broca	Severe	30	81
24	IS	sc	IFG	M	62	12	24	AAT	Broca	Moderate	55	95
25	IS	a/sc	IFG	M	60	9	85	AAT	Wernicke	Moderate	5	64
26	IS	a	IFG	F	83	13	31	AAT	Wernicke	Moderate	50	84
27	IS	a/sc	IFG	M	51	16	54	AAT	Wernicke	Moderate	0	90
28	IS	a/sc	IFG	M	80	20	34	BIAS-R	Subacute Aph.	Severe **^3^**	−75	78
29	IHS	sc	IFG	M	67	13	92	BIAS-R	Subacute Aph.	Severe	−70	47
30	IS	a/sc/p	M1	M	55	16	99	BIAS_R	Subacute Aph.	Severe	−20	44
31	IHS	a/sc	FPC	F	54	17	135	BIAS-R	Subacute Aph.	Severe	−70	57
32	IS	a/sc	IFG	F	65	10	33	BIAS-R	Subacute Aph.	Severe	−60	38
33	IS	a/sc	IFG	M	57	17	43	BIAS-R	Subacute Aph.	Severe	−60	54
34	IS	a/p/sc	M1	M	54	13	50	BIAS-R	Subacute Aph.	Severe	−125	48
35	IS	a/sc	FPC	F	38	13	144	BIAS-R	Subacute Aph.	Severe	−65	55
36	IHS	a/sc	IFG	F	65	17	51	BIAS-R	Subacute Aph.	Severe	−65	55
37	IS	a/p/sc	M1	F	48	10	85	BIAS-R	Subacute Aph.	Severe	40	90

IS = Ischemic stroke; IHS = Intracerebral hemorrhagic stroke; IFG = Inferior frontal gyrus; M1 = Motor cortex; FPC = Frontopolar cortex; F = Female; M = Male; ^1^ lesion location (see Figure A3 Appendix D): a = anterior part of left MCA territory; p = posterior part of the left MCA territory; sc = subcortical part of left MCA territory; ^2^ aphasia diagnosis according to Aachen Aphasia Test (AAT)/BIAS-R does not classify into aphasia types; ^3^ severity according to AAT profile level value; ^4^ severity according to BIAS-R percentile rank (PR) and stanine; ^5^ initial severity at the beginning of rehabilitation stay: Early Rehabilitation Barthel Index (ERBI); ^6^ initial severity at the beginning of rehabilitation stay: Functional Independence Measure (FIM).

**Table A4:** Language data for AAT sample at all three assessments: T1, T2, T3.

PwA	Spontaneous Speech Ratings			Profile Level			Token Test ^1^ (Errors)			Repetition ^1^			Written Language ^1^			Naming ^1^			Comprehension ^1^		
	(0–5)			(T-Norm)			(Max. 50)			(Max. 150)			(Max. 90)			(Max. 120)			(Max. 120)		
	T1	T2	T3	T1	T2	T3	T1	T2	T3	T1	T2	T3	T1	T2	T3	T1	T2	T3	T1	T2	T3
1	2 5 4 3 4 4	3 5 4 3 4 4	3 5 5 3 4 4	52.22	52.88	56.33	34	34	28	138	143	144	39	44	59	66	66	94	94	81	96
2	2 4 5 3 4 4	2 5 4 3 4 4	3 5 5 3 4 4	57.13	58.80	61.39	16	12	8	140	140	144	76	78	82	82	91	98	82	92	93
3	2 5 4 3 2 4	2 5 4 3 2 4	2 5 5 3 2 4	44.70	44.83	47.13	27	23	13	49	45	51	30	36	44	30	24	41	75	81	94
4	3 3 5 3 2 4	3 3 5 3 2 4	4 4 5 4 3 4	60.53	61.68	64.25	5	0	0	136	133	142	73	76	78	107	107	108	99	106	110
5	3 4 5 3 3 4	3 4 5 3 2 4	3 4 5 4 3 4	55.80	62.39	67.27	25	3	0	133	140	146	72	84	86	96	96	109	93	104	104
6	3 5 5 4 4 4	3 5 5 4 5 4	4 5 5 4 5 5	63.70	67.55	73.17	4	0	0	146	147	149	87	87	89	83	100	112	102	109	113
7	2 2 4 3 2 2	2 2 4 3 2 2	2 2 4 3 2 4	47.71	47.66	49.14	30	32	24	103	105	109	28	27	28	66	59	76	76	76	77
8	2 5 4 3 4 4	2 5 4 3 4 4	3 5 5 3 5 4	54.43	54.93	56.82	32	34	28	137	143	142	71	67	76	76	76	87	81	90	96
9	4 5 5 3 4 4	3 5 4 3 4 4	4 5 5 4 4 4	49.31	50.63	54.29	11	8	5	76	98	113	38	39	50	70	78	87	104	93	112
10	1 1 0 0 0 0	1 1 0 0 0 1	1 2 0 3 2 1	40.17	43.58	45.56	33	29	20	15	38	66	14	21	29	0	16	30	79	91	81
11	1 4 4 1 1 0	1 4 4 1 1 0	1 4 4 1 1 0	40.91	42.20	44.22	46	41	37	43	54	79	9	14	22	16	9	18	67	72	81
12	1 0 0 0 0 0	2 3 4 2 2 2	2 3 4 3 2 2	39.95	43.73	48.41	50	46	40	65	86	109	19	37	47	2	14	52	61	54	82
13	3 3 5 3 2 3	3 3 5 3 2 3	4 3 4 4 4 4	58.34	60.33	65.73	2	0	0	127	128	144	61	71	78	106	106	113	88	101	107
14	1 2 4 3 2 0	2 2 4 3 2 1	3 3 5 3 2 4	45.44	50.28	53.46	41	22	9	77	100	112	30	39	54	47	88	92	89	94	105
15	2 5 3 2 3 3	2 5 3 2 3 3	2 5 3 2 3 3	45.28	47.13	48.99	44	35	27	114	116	118	7	14	25	43	57	47	47	57	76
16	1 4 3 3 2 1	1 4 4 3 2 1	2 4 5 3 2 2	46.73	47.44	49.51	44	34	22	120	120	122	20	29	27	38	28	48	71	64	71
17	2 4 4 3 3 3	2 4 4 3 2 3	2 4 4 3 2 3	57.97	57.40	61.57	1	2	2	134	134	141	74	69	82	74	79	86	106	98	109
18	1 4 4 3 4 1	1 5 4 3 4 1	1 5 4 3 4 1	43.20	41.78	43.55	46	45	43	104	100	106	6	7	8	11	0	1	66	62	62
19	4 3 5 4 4 5	4 3 4 4 4 5	5 4 5 5 4 5	61.01	63.79	69.80	0	0	0	124	124	145	76	81	84	109	115	119	103	110	115
20	1 1 3 3 2 2	2 1 3 3 2 2	2 1 3 3 2 2	44.24	43.58	45.42	38	43	39	89	89	103	9	8	11	33	31	41	55	60	76
21	1 1 4 3 2 0	1 1 4 3 2 1	2 1 5 3 2 2	49.67	50.40	54.75	15	12	3	100	103	117	42	73	60	71	73	92	67	75	80
22	1 5 2 1 3 3	1 5 2 1 2 3	1 5 3 1 2 3	37.87	40.19	42.24	43	41	39	20	34	50	0	4	11	10	19	24	53	55	62
23	1 3 4 3 2 1	1 3 4 3 2 1	2 3 4 2 3 1	45.43	45.91	48.60	31	36	30	85	79	101	31	37	41	14	39	47	84	87	101
24	3 3 5 3 3 2	3 3 5 3 3 2	3 3 5 3 3 4	50.72	52.43	56.21	15	13	2	101	109	117	51	58	70	77	85	91	86	84	97
25	2 4 3 3 3 3	2 4 3 3 3 3	2 4 3 3 3 3	47.03	46.68	47.98	45	36	38	136	138	139	7	7	6	3	0	12	62	57	53
26	2 4 3 3 4 3	2 5 3 3 4 3	2 5 3 3 4 3	49.41	48.53	50.47	39	41	39	135	132	133	48	47	59	17	11	25	48	46	77
27	2 4 4 3 2 3	2 4 5 3 2 3	3 4 5 3 2 3	50.25	51.22	54.42	33	27	17	126	133	139	47	44	59	50	64	63	50	50	76

^1^ Raw scores.

**Table A5:** Language data for BIAS-R sample at all three assessments: T1, T2, T3.

PwA	Profile Level Mean Percentage Value			ASV ^1^ Auditory Comprehension			AUT ^1^ Automatic Language			ELIZ ^1^ Naming			WFL ^2^ Verbal Fluency			NACH ^1^ Repetition			LSV ^1^ Reading Comprehension			LLES ^1^ Reading Aloud			SCHR ^1^ Writing		
	(T-Norm)			(Max. 72)			(Max. 36)			(Max. 36)			(Max. 15)			(Max. 36)			(Max. 72)			(Max. 36)			(Max. 18)		
	T1	T2	T3	T1	T2	T3	T1	T2	T3	T1	T2	T3	T1	T2	T3	T1	T2	T3	T1	T2	T3	T1	T2	T3	T1	T2	T3
1	31.5	35.5	38.5	41	42	46	13	22	27	16	15	17	0	0	0				10	10	14	4	9	13	0	0	0
2	41	41	41.5	57	51	51	12	18	26	21	19	14	0	0	0	12	14	14	39	43	50	23	23	22	0	0	0
3	34	36	40.5	46	52	54	11	12	22	14	15	20	0	0	0	2	4	14	47	52	60	0	0	0	0	1	2
4	41.5	43.5	44.5	63	62	65	23	30	32	12	18	20	0	0	1	20	25	28	53	58	61	6	11	12	5	6	6
5	35	40	44.5	35	49	54	18	23	26	14	13	16	0	0	0	4	4	34	29	41	46	6	8	25	0	11	11
6	43.5	44	47	50	62	61	28	28	33	14	17	21	0	0	1	30	31	35	47	48	57	27	28	31	2	2	6
7	35.5	37	41.5	44	44	48	12	14	17	15	11	18	0	0	0	7	12	18	42	40	51	0	10	16	1	1	3
8	35	37	40.5	34	42	51	12	14	21	13	14	16	0	0	0	6	7	15	47	55	51	3	6	2	1	1	6
9	38.5	39.5	42.5	48	59	65	12	11	12	13	12	16	0	0	0	4	6	15	57	61	64	5	6	9	6	6	12
10	38.5	40	42.5	42	39	52	14	16	25	15	15	16	0	0	0	9	14	22	50	50	47	15	19	20	0	2	4

^1^ Raw score; ^2^ scoring according to BIAS-R test manual.

**Table A6:** Individually rated side effects per therapy session (TH1–TH10); 1 = no side effect to 10 = strong side effect.

PwA	Stim	Th1	Th2	Th3	Th4	Th5	Th6	Th7	Th8	Th9	Th10
	IFG ^1^	3	2	1	1	1	1	1	2	1	1
2	IFG	0	9	2	2	2	2	2	2	1	1
3	IFG	2	2	1	1	1	1	1	1	1	1
4	M1 ^2^	0	0	0	0	0	0	0	0	0	0
5	FPC ^3^	5	5	3	3	2	2	2	2	2	2
6	IFG	0	0	1	0	1	0	1	0	1	0
7	IFG	0	0	0	0	0	0	0	0	0	0
8	IFG	4	0	0	0	0	0	0	0	0	0
9	IFG	0	0	0	0	0	0	0	0	0	0
10	IFG	2	2	2	2	2	0	0	2	2	0
11	M1	0	0	0	1	0	0	0	1	0	0
12	IFG	2	2	2	1	1	1	1	1	1	1
13	IFG	2	1	2	1	1	1	0	1	1	0
14	IFG	6	4	5	4	4	3	2	4	2	2
15	FPC	4	3	3	3	3	2	2	3	2	2
16	IFG	1	1	1	1	1	1	1	1	1	1
17	IFG	0	0	0	0	0	0	0	0	0	0
18	IFG	4	4	4	4	5	5	5	5	5	6
19	IFG	2	2	1	1	1	1	1	1	0	1
20	IFG	0	0	0	0	0	0	0	0	0	0
21	IFG	8	7	7	6	6	6	7	7	6	7
22	IFG	0	0	0	0	0	0	0	0	0	0
23	IFG	3	2	3	1	1	2	2	2	0	2
24	FPC	2	2	2	2	1	1	1	1	1	1
25	IFG	2	2	2	2	2	2	2	2	2	2
26	M1	0	0	0	0	0	0	0	0	0	0
27	IFG	4	2	2	2	2	2	2	1	1	1
28	IFG	6	6	6	5	6	6	7	6	6	6
29	IFG	0	0	0	0	0	0	0	0	0	0
30	IFG	6	5	6	3	4	3	3	3	3	3
31	IFG	6	7	7	7	6	8	6	6	6	7
32	IFG	3	3	1	1	1	2	2	3	2	3
33	IFG	0	0	0	0	0	0	0	0	0	0
34	IFG	0	0	0	0	0	0	0	0	0	0
35	IFG	4	2	0	2	0	0	0	0	0	0
36	IFG	2	2	1	2	3	2	1	2	4	4
37	IFG	0	0	0	0	0	1	0	0	0	0

^1^ Inferior frontal gyrus; ^2^ primary motor cortex; ^3^ frontopolar cortex.

## References

[1] Mitchell C., Gittins M., Tyson S., Vail A., Conroy P., Paley L., Bowen A. (2021). Prevalence of aphasia and dysarthria among inpatient stroke survivors: Describing the population, therapy provision and outcomes on discharge. Aphasiology.

[2] Engelter S.T., Gostynski M., Papa S., Frei M., Born C., Ajdacic-Gross V., Gutzwiller F., Lyrer P.A. (2006). Epidemiology of aphasia attributable to first ischemic stroke: Incidence, severity, fluency, etiology, and thrombolysis. Stroke.

[3] Berthier M.L. (2005). Poststroke aphasia : Epidemiology, pathophysiology and treatment. Drugs Aging.

[4] Hilari K., Cruice M., Sorin-Peters R., Worrall L. (2015). Quality of Life in Aphasia: State of the Art. Folia Phoniatr. Logop. Off. Organ Int. Assoc. Logop. Phoniatr. (IALP).

[5] Hilari K., Byng S. (2009). Health-related quality of life in people with severe aphasia. Int. J. Lang. Commun. Disord..

[6] Stahl B., Millrose S., Denzler P., Lucchese G., Jacobi F., Flöel A. (2022). Intensive Social Interaction for Treatment of Poststroke Depression in Subacute Aphasia: The CONNECT Trial. Stroke.

[7] Baker C., Worrall L., Rose M., Hudson K., Ryan B., O’Byrne L. (2018). A systematic review of rehabilitation interventions to prevent and treat depression in post-stroke aphasia. Disabil. Rehabil..

[8] Breitenstein C., Grewe T., Flöel A., Ziegler W., Springer L., Martus P., Huber W., Willmes K., Ringelstein E.B., Haeusler K.G. (2017). Intensive speech and language therapy in patients with chronic aphasia after stroke: A randomised, open-label, blinded-endpoint, controlled trial in a health-care setting. Lancet.

[9] Brady M.C., Kelly H., Godwin J., Enderby P., Campbell P. (2016). Speech and language therapy for aphasia following stroke. Cochrane Database Syst. Rev..

[10] Kiran S., Thompson C.K. (2019). Neuroplasticity of Language Networks in Aphasia: Advances, Updates, and Future Challenges. Front. Neurol..

[11] Husak R.S., Wallace S.E., Marshall R.C., Visch-Brink E.G. (2023). A systematic review of aphasia therapy provided in the early period of post-stroke recovery. Aphasiology.

[12] Eley E., van den Berg M., Rose M.L., Pierce J.E., Foster A., Lamborn E., D’Souza S., Godecke E., Lanyon L., Shiggins C. (2023). The effects of cognitive-linguistic interventions to treat aphasia in the first 90 days post-stroke: A systematic review. Aphasiology.

[13] Godecke E., Armstrong E., Rai T., Ciccone N., Rose M.L., Middleton S., Whitworth A., Holland A., Ellery F., Hankey G.J. (2021). A randomized control trial of intensive aphasia therapy after acute stroke: The Very Early Rehabilitation for SpEech (VERSE) study. Int. J. Stroke.

[14] Ding X., Zhang S., Huang W., Zhang S., Zhang L., Hu J., Li J., Ge Q., Wang Y., Ye X. (2022). Comparative efficacy of non-invasive brain stimulation for post-stroke aphasia: A network meta-analysis and meta-regression of moderators. Neurosci. Biobehav. Rev..

[15] Kielar A., Patterson D., Chou Y. (2022). Efficacy of repetitive transcranial magnetic stimulation in treating stroke aphasia: Systematic review and meta-analysis. Clin. Neurophysiol. Off. J. Int. Fed. Clin. Neurophysiol..

[16] Elsner B., Kugler J., Mehrholz J. (2020). Transcranial direct current stimulation (tDCS) for improving aphasia after stroke: A systematic review with network meta-analysis of randomized controlled trials. J. NeuroEng. Rehabil..

[17] Nitsche M.A., Paulus W. (2001). Sustained excitability elevations induced by transcranial DC motor cortex stimulation in humans. Neurology.

[18] Thiel A., Zumbansen A. (2016). The pathophysiology of post-stroke aphasia: A network approach. Restor. Neurol. Neurosci..

[19] Harvey D.Y., Hamilton R. (2022). Noninvasive brain stimulation to augment language therapy for poststroke aphasia. Handb. Clin. Neurol..

[20] Biou E., Cassoudesalle H., Cogné M., Sibon I., de Gabory I., Dehail P., Aupy J., Glize B. (2019). Transcranial direct current stimulation in post-stroke aphasia rehabilitation: A systematic review. Ann. Phys. Rehabil. Med..

[21] Meinzer M., Darkow R., Lindenberg R., Flöel A. (2016). Electrical stimulation of the motor cortex enhances treatment outcome in post-stroke aphasia. Brain.

[22] Branscheidt M., Hoppe J., Zwitserlood P., Liuzzi G. (2018). tDCS over the motor cortex improves lexical retrieval of action words in poststroke aphasia. J. Neurophysiol..

[23] Hamilton R.H., Chrysikou E.G., Coslett B. (2011). Mechanisms of aphasia recovery after stroke and the role of noninvasive brain stimulation. Brain Lang..

[24] Hamilton R.H. (2016). Neuroplasticity in the language system: Reorganization in post-stroke aphasia and in neuromodulation interventions. Restor. Neurol. Neurosci..

[25] Shah-Basak P., Boukrina O., Li X.R., Jebahi F., Kielar A. (2023). Targeted neurorehabilitation strategies in post-stroke aphasia. Restor. Neurol. Neurosci..

[26] Marangolo P. (2020). The potential effects of transcranial direct current stimulation (tDCS) on language functioning: Combining neuromodulation and behavioral intervention in aphasia. Neurosci. Lett..

[27] Vines B.W., Norton A.C., Schlaug G. (2011). Non-invasive brain stimulation enhances the effects of melodic intonation therapy. Front. Psychol..

[28] Spielmann K., van de Sandt-Koenderman W.M.E., Heijenbrok-Kal M.H., Ribbers G.M. (2018). Transcranial Direct Current Stimulation Does Not Improve Language Outcome in Subacute Poststroke Aphasia. Stroke.

[29] Stockbridge M.D., Elm J., Breining B.L., Tippett D.C., Sebastian R., Cassarly C., Teklehaimanot A., Spell L.A., Sheppard S.M., Vitti E. (2023). Transcranial Direct-Current Stimulation in Subacute Aphasia: A Randomized Controlled Trial. Stroke.

[30] You D.S., Kim D.-Y., Chun M.H., Jung S.E., Park S.J. (2011). Cathodal transcranial direct current stimulation of the right Wernicke’s area improves comprehension in subacute stroke patients. Brain Lang..

[31] Hamilton R.H., Kessler S.K., Castillo-Saavedra L., Fregni F., Martin D., Loo C., Knotkova H., Woods A.J., Knotkova H. (2019). Methodological Considerations for Transcranial Direct Current Stimulation in Clinical Trials. Practical Guide to Transcranial Direct Current Stimulation: Principles, Procedures and Applications.

[32] Samanta D., Landes S.J. (2021). Implementation Science to Improve Quality of Neurological Care. Pediatr. Neurol..

[33] Juckett L.A., Wengerd L.R., Faieta J., Griffin C.E. (2020). Evidence-Based Practice Implementation in Stroke Rehabilitation: A Scoping Review of Barriers and Facilitators. Am. J. Occup. Ther. Off. Publ. Am. Occup. Ther. Assoc..

[34] van Stan J.H., Holmes J., Wengerd L., Juckett L.A., Whyte J., Pinto S.M., Katz L.W., Wolfberg J. (2023). Rehabilitation Treatment Specification System: Identifying Barriers, Facilitators, and Strategies for Implementation in Research, Education, and Clinical Care. Arch. Phys. Med. Rehabil..

[35] Douglas N.F., Feuerstein J.L., Oshita J.Y., Schliep M.E., Danowski M.L. (2022). Implementation Science Research in Communication Sciences and Disorders: A Scoping Review. Am. J. Speech-Lang. Pathol..

[36] Biniek R., Huber W., Glindemann R., Willmes K., Klumm H. (1992). Der Aachener Aphasie-Bedside-Test—Testpsychologische Gütekriterien. Nervenarzt.

[37] Huber W., Poeck K., Willmes K. (1984). The Aachen Aphasia Test. Adv. Neurol..

[38] Peitz D., Schumann-Werner B., Hussmann K., Pinho J., Chen H., Binkofski F., Huber W., Willmes K., Heim S., Schulz J.B. (2024). Success rates of intensive aphasia therapy: Real-world data from 448 patients between 2003 and 2020. J. Neurol..

[39] Shah-Basak P.P., Norise C., Garcia G., Torres J., Faseyitan O., Hamilton R.H. (2015). Individualized treatment with transcranial direct current stimulation in patients with chronic non-fluent aphasia due to stroke. Front. Hum. Neurosci..

[40] de Aguiar V., Bastiaanse R., Capasso R., Gandolfi M., Smania N., Rossi G., Miceli G. (2015). Can tDCS enhance item-specific effects and generalization after linguistically motivated aphasia therapy for verbs?. Front. Behav. Neurosci..

[41] Bernhardt J., Hayward K.S., Kwakkel G., Ward N.S., Wolf S.L., Borschmann K., Krakauer J.W., Boyd L.A., Carmichael S.T., Corbett D. (2017). Agreed definitions and a shared vision for new standards in stroke recovery research: The Stroke Recovery and Rehabilitation Roundtable taskforce. Int. J. Stroke.

[42] Salmaso D., Longoni A.M. (1985). Problems in the assessment of hand preference. Cortex.

[43] Willmes K., Poeck K., Willmes K., Poeck K. (1984). Ergebnisse einer multizentrischen untersuchung über die spontanprognose von aphasien vaskulärer ätiologie. Nervenarzt.

[44] Luzzatti C., de Bleser R., Scola I., Frustaci M., Willmes K. (2023). Update on the psychometric properties for the Italian version of the Aachen Aphasia Test (IT-AAT). Aphasiology.

[45] Willmes K. (1985). An approach to analyzing a single subject’s scores obtained in a standardized test with application to the Aachen Aphasia Test (AAT). J. Clin. Exp. Neuropsychol..

[46] Richter K., Hielscher-Fastabend M. (2018). BIAS A&R: Bielefelder Aphasie Screening Akut und Reha.

[47] Rollnik J.D. (2011). The Early Rehabilitation Barthel Index (ERBI). Die Rehabil..

[48] Granger C.V., Hamilton B.B., Linacre J.M., Heinemann A.W., Wright B.D. (1993). Performance profiles of the functional independence measure. Am. J. Phys. Med. Rehabil..

[49] Fregni F., Nitsche M.A., Loo C.K., Brunoni A.R., Marangolo P., Leite J., Carvalho S., Bolognini N., Caumo W., Paik N.J. (2015). Regulatory Considerations for the Clinical and Research Use of Transcranial Direct Current Stimulation (tDCS): Review and recommendations from an expert panel. Clin. Res. Regul. Aff..

[50] Friederici A.D. (2011). The brain basis of language processing: From structure to function. Physiol. Rev..

[51] Datta A., Baker J.M., Bikson M., Fridriksson J. (2011). Individualized model predicts brain current flow during transcranial direct-current stimulation treatment in responsive stroke patient. Brain Stimul..

[52] Pulvermüller F., Fadiga L. (2010). Active perception: Sensorimotor circuits as a cortical basis for language. Nat. Rev. Neurosci..

[53] Datta A., Bikson M., Fregni F. (2010). Transcranial direct current stimulation in patients with skull defects and skull plates: High-resolution computational FEM study of factors altering cortical current flow. NeuroImage.

[54] Nitsche M.A., Doemkes S., Karaköse T., Antal A., Liebetanz D., Lang N., Tergau F., Paulus W. (2007). Shaping the effects of transcranial direct current stimulation of the human motor cortex. J. Neurophysiol..

[55] van Hees S., McMahon K., Angwin A., de Zubicaray G., Read S., Copland D.A. (2014). A functional MRI study of the relationship between naming treatment outcomes and resting state functional connectivity in post-stroke aphasia. Hum. Brain Mapp..

[56] Thompson C.K., den Ouden D.-B. (2008). Neuroimaging and recovery of language in aphasia. Curr. Neurol. Neurosci. Rep..

[57] Hickey J., Shrubsole K., Worrall P.L., Power E. (2019). Implementing aphasia recommendations in the acute setting: Speech-language pathologists’ perspectives of a behaviour change intervention. Aphasiology.

[58] Brown S.E., Brady M.C., Worrall L., Scobbie L. (2021). A narrative review of communication accessibility for people with aphasia and implications for multi-disciplinary goal setting after stroke. Aphasiology.

[59] Springer L., Papathanasiou I., de Bleser R. (2003). Chapter 10—Reduced Syntax Therapy (REST)-A Compensatory Approach to Agrammatism. The Sciences of Aphasia.

[60] JAS 24. JASP Team (2024). JASP (Version 0.18.3)[Computer software].

[61] Faul F., Erdfelder E., Buchner A., Lang A.-G. (2009). Statistical power analyses using G*Power 3.1: Tests for correlation and regression analyses. Behav. Res. Methods.

[62] Holm S. (1979). A simple sequentially rejective multiple test procedure. Scand. J. Statist..

[63] Morey R.D. (2008). Confidence Intervals from Normalized Data: A correction to Cousineau (2005). Tutor. Quant. Methods Psychol..

[64] Cohen J. (2013). Statistical Power Analysis for the Behavioral Sciences.

[65] Lakens D. (2013). Calculating and reporting effect sizes to facilitate cumulative science: A practical primer for *t*-tests and ANOVAs. Front. Psychol..

[66] Shrout P.E., Fleiss J.L. (1979). Intraclass correlations: Uses in assessing rater reliability. Psychol. Bull..

[67] Kristinsson S., Basilakos A., den Ouden D.B., Cassarly C., Spell L.A., Bonilha L., Rorden C., Hillis A.E., Hickok G., Johnson L. (2023). Predicting Outcomes of Language Rehabilitation: Prognostic Factors for Immediate and Long-Term Outcomes After Aphasia Therapy. J. Speech Lang. Hear. Res..

[68] Plowman E., Hentz B., Ellis C. (2012). Post-stroke aphasia prognosis: A review of patient-related and stroke-related factors. J. Eval. Clin. Pract..

[69] Watila M.M., Balarabe S.A. (2015). Factors predicting post-stroke aphasia recovery. J. Neurol. Sci..

[70] Wilson S.M., Entrup J.L., Schneck S.M., Onuscheck C.F., Levy D.F., Rahman M., Willey E., Casilio M., Yen M., Brito A.C. (2023). Recovery from aphasia in the first year after stroke. Brain.

[71] Wilmskoetter J., He X., Caciagli L., Jensen J.H., Marebwa B., Davis K.A., Fridriksson J., Basilakos A., Johnson L.P., Rorden C. (2022). Language Recovery after Brain Injury: A Structural Network Control Theory Study. J. Neurosci..

[72] Marangolo P., Fiori V., Sabatini U., de Pasquale G., Razzano C., Caltagirone C., Gili T. (2016). Bilateral Transcranial Direct Current Stimulation Language Treatment Enhances Functional Connectivity in the Left Hemisphere: Preliminary Data from Aphasia. J. Cogn. Neurosci..

[73] DeDe G., Hoover E. (2021). Measuring Change at the Discourse-Level Following Conversation Treatment. Top. Lang. Disord..

[74] Hoover E.L., Caplan D., Waters G., Budson A. (2015). Effects of impairment-based individual and socially oriented group therapies on verb production in aphasia. Aphasiology.

[75] Nicholas L.E., Brookshire R.H. (1993). A system for quantifying the informativeness and efficiency of the connected speech of adults with aphasia. J. Speech Hear. Res..

[76] Harvey S., Stone M., Zingelman S., Copland D.A., Kilkenny M.F., Godecke E., Cadilhac D.A., Kim J., Olaiya M.T., Rose M.L. (2024). Comprehensive quality assessment for aphasia rehabilitation after stroke: Protocol for a multicentre, mixed-methods study. BMJ Open.

[77] Hilari K., Byng S., Lamping D.L., Smith S.C. (2003). Stroke and Aphasia Quality of Life Scale-39 (SAQOL-39): Evaluation of acceptability, reliability, and validity. Stroke.

[78] Stahl B., Henseler I., Turner R., Geyer S., Kotz S.A. (2013). How to engage the right brain hemisphere in aphasics without even singing: Evidence for two paths of speech recovery. Front. Hum. Neurosci..

[79] Stockert A., Wawrzyniak M., Klingbeil J., Wrede K., Kümmerer D., Hartwigsen G., Kaller C.P., Weiller C., Saur D. (2020). Dynamics of language reorganization after left temporo-parietal and frontal stroke. Brain.

[80] Saur D., Lange R., Baumgaertner A., Schraknepper V., Willmes K., Rijntjes M., Weiller C. (2006). Dynamics of language reorganization after stroke. Brain.

[81] Heiss W.-D., Thiel A. (2006). A proposed regional hierarchy in recovery of post-stroke aphasia. Brain Lang..

[82] Saur D., Hartwigsen G. (2012). Neurobiology of language recovery after stroke: Lessons from neuroimaging studies. Arch. Phys. Med. Rehabil..

[83] Galletta E.E., Conner P., Vogel-Eyny A., Marangolo P. (2016). Use of tDCS in Aphasia Rehabilitation: A Systematic Review of the Behavioral Interventions Implemented With Noninvasive Brain Stimulation for Language Recovery. Am. J. Speech-Lang. Pathol..

[84] Stahl B., Darkow R., von Podewils V., Meinzer M., Grittner U., Reinhold T., Grewe T., Breitenstein C., Flöel A. (2019). Transcranial Direct Current Stimulation to Enhance Training Effectiveness in Chronic Post-Stroke Aphasia: A Randomized Controlled Trial Protocol. Front. Neurol..

[85] Saur D., Kreher B.W., Schnell S., Kümmerer D., Kellmeyer P., Vry M.-S., Umarova R., Musso M., Glauche V., Abel S. (2008). Ventral and dorsal pathways for language. Proc. Natl. Acad. Sci. USA.

[86] Hickok G., Poeppel D. (2007). The cortical organization of speech processing. Nat. Reviews. Neurosci..

[87] Kümmerer D., Hartwigsen G., Kellmeyer P., Glauche V., Mader I., Klöppel S., Suchan J., Karnath H.-O., Weiller C., Saur D. (2013). Damage to ventral and dorsal language pathways in acute aphasia. Brain.

[88] Hartwigsen G., Volz L.J. (2021). Probing rapid network reorganization of motor and language functions via neuromodulation and neuroimaging. NeuroImage.

[89] Boros K., Poreisz C., Münchau A., Paulus W., Nitsche M.A. (2008). Premotor transcranial direct current stimulation (tDCS) affects primary motor excitability in humans. Eur. J. Neurosci..

[90] Stagg C.J., O’Shea J., Kincses Z.T., Woolrich M., Matthews P.M., Johansen-Berg H. (2009). Modulation of movement-associated cortical activation by transcranial direct current stimulation. Eur. J. Neurosci..

[91] Peña-Gómez C., Sala-Lonch R., Junqué C., Clemente I.C., Vidal D., Bargalló N., Falcón C., Valls-Solé J., Pascual-Leone Á., Bartrés-Faz D. (2012). Modulation of large-scale brain networks by transcranial direct current stimulation evidenced by resting-state functional MRI. Brain Stimul..

[92] Darkow R., Martin A., Würtz A., Flöel A., Meinzer M. (2017). Transcranial direct current stimulation effects on neural processing in post-stroke aphasia. Hum. Brain Mapp..

[93] Dreyer F.R., Doppelbauer L., Büscher V., Arndt V., Stahl B., Lucchese G., Hauk O., Mohr B., Pulvermüller F. (2021). Increased Recruitment of Domain-General Neural Networks in Language Processing Following Intensive Language-Action Therapy: fMRI Evidence From People With Chronic Aphasia. Am. J. Speech-Lang. Pathol..

[94] Richardson J., Datta A., Dmochowski J., Parra L.C., Fridriksson J. (2015). Feasibility of using high-definition transcranial direct current stimulation (HD-tDCS) to enhance treatment outcomes in persons with aphasia. NeuroRehabilitation.

[95] Fridriksson J., Rorden C., Elm J., Sen S., George M.S., Bonilha L. (2018). Transcranial Direct Current Stimulation vs Sham Stimulation to Treat Aphasia After Stroke: A Randomized Clinical Trial. JAMA Neurol..

[96] Shah-Basak P.P., Sivaratnam G., Teti S., Francois-Nienaber A., Yossofzai M., Armstrong S., Nayar S., Jokel R., Meltzer J. (2020). High definition transcranial direct current stimulation modulates abnormal neurophysiological activity in post-stroke aphasia. Sci. Rep..

[97] Zumbansen A., Black S.E., Chen J.L., J Edwards D., Hartmann A., Heiss W.-D., Lanthier S., Lesperance P., Mochizuki G., Paquette C. (2020). Non-invasive brain stimulation as add-on therapy for subacute post-stroke aphasia: A randomized trial (NORTHSTAR). Eur. Stroke J..

[98] Curran G.M., Bauer M., Mittman B., Pyne J.M., Stetler C. (2012). Effectiveness-implementation hybrid designs: Combining elements of clinical effectiveness and implementation research to enhance public health impact. Med. Care.

[99] Morris Z.S., Wooding S., Grant J. (2011). The answer is 17 years, what is the question: Understanding time lags in translational research. J. R. Soc. Med..

[100] Morris J.H., Bernhardsson S., Bird M.-L., Connell L., Lynch E., Jarvis K., Kayes N.M., Miller K., Mudge S., Fisher R. (2020). Implementation in rehabilitation: A roadmap for practitioners and researchers. Disabil. Rehabil..

[101] Lefaucheur J.-P., Antal A., Ayache S.S., Benninger D.H., Brunelin J., Cogiamanian F., Cotelli M., de Ridder D., Ferrucci R., Langguth B. (2017). Evidence-based guidelines on the therapeutic use of transcranial direct current stimulation (tDCS). Clin. Neurophysiol. Off. J. Int. Fed. Clin. Neurophysiol..

[102] Cohen Kadosh R., Soskic S., Iuculano T., Kanai R., Walsh V. (2010). Modulating neuronal activity produces specific and long-lasting changes in numerical competence. Curr. Biol. CB.

[103] Meinzer M., Jähnigen S., Copland D.A., Darkow R., Grittner U., Avirame K., Rodriguez A.D., Lindenberg R., Flöel A. (2014). Transcranial direct current stimulation over multiple days improves learning and maintenance of a novel vocabulary. Cortex A J. Devoted Study Nerv. Syst. Behav..

[104] Reis J., Schambra H.M., Cohen L.G., Buch E.R., Fritsch B., Zarahn E., Celnik P.A., Krakauer J.W. (2009). Noninvasive cortical stimulation enhances motor skill acquisition over multiple days through an effect on consolidation. Proc. Natl. Acad. Sci. USA.

[105] Campana S., Caltagirone C., Marangolo P. (2015). Combining Voxel-based Lesion-symptom Mapping (VLSM) with A-tDCS Language Treatment: Predicting Outcome of Recovery in Nonfluent Chronic Aphasia. Brain Stimul..

[106] Fridriksson J., Richardson J.D., Baker J.M., Rorden C. (2011). Transcranial direct current stimulation improves naming reaction time in fluent aphasia: A double-blind, sham-controlled study. Stroke.

